# Multi-Scale In Vivo Systems Analysis Reveals the Influence of Immune Cells on TNF-α-Induced Apoptosis in the Intestinal Epithelium

**DOI:** 10.1371/journal.pbio.1001393

**Published:** 2012-09-25

**Authors:** Ken S. Lau, Virna Cortez-Retamozo, Sarah R. Philips, Mikael J. Pittet, Douglas A. Lauffenburger, Kevin M. Haigis

**Affiliations:** 1 Molecular Pathology Unit, Massachusetts General Hospital, Charlestown, Massachusetts, United States of America; 2 Center for Cancer Research, Massachusetts General Hospital, Charlestown, Massachusetts, United States of America; 3 Center for Systems Biology, Massachusetts General Hospital, Boston, Massachusetts, United States of America; 4 Department of Pathology, Harvard Medical School, Boston, Massachusetts, United States of America; 5 Department of Biological Engineering, Massachusetts Institute of Technology, Cambridge, Massachusetts, United States of America; 6 Koch Institute for Integrative Cancer Research, Massachusetts Institute of Technology, Cambridge, Massachusetts, United States of America; University of Alabama at Birmingham, United States of America

## Abstract

Intestinal epithelial cells exist within a complex environment that affects how they interpret and respond to stimuli. We have applied a multi-scale in vivo systems approach to understand how intestinal immune cells communicate with epithelial cells to regulate responses to inflammatory signals. Multivariate modeling analysis of a large dataset composed of phospho-signals, cytokines, and immune cell populations within the intestine revealed an intimate relationship between immune cells and the epithelial response to TNF-α. Ablation of lymphocytes in the intestine prompted a decrease in the expression of MCP-1, which in turn increased the steady state number of intestinal plasmacytoid dendritic cells (pDCs). This change in the immune compartment affected the intestinal cytokine milieu and subsequent epithelial cell signaling network, with cells becoming hypersensitive to TNF-α-induced apoptosis in a way that could be predicted by mathematical modeling. In summary, we have uncovered a novel cellular network that regulates the response of intestinal epithelial cells to inflammatory stimuli in an in vivo setting.

## Abstract 2

An intercellular communication network that controls intestinal homeostasis in animals treated acutely with the pro-inflammatory cytokine TNF-α is uncovered by multi-scale systems analysis.

## Introduction

Tumor necrosis factor alpha (TNF-α) is a pro-inflammatory cytokine that operates through a pleiotropic signaling network to influence a diverse array of cellular behaviors, including the induction of apoptosis, survival, proliferation, cell cycle arrest, or differentiation [1]. TNF-α primarily signals through the ubiquitously expressed receptor TNFR1, with TNFR2 playing an accessory role. Upon binding to ligand, the death domain of TNFR1 recruits TRADD, RIP1, TRAF2/5, and cIAP1/2, which together constitute complex I at the cell surface [2]. Complex I is thought to primarily play a pro-survival role by activating downstream NFκB and MAPK pathways [3]. Upon internalization of this complex, cIAP1/2 auto-degradation triggers the release of RIP, which forms complex 2 with FADD and pro-caspase 8 [4]. In conjunction with the down-regulation of c-Flip, complex 2 activates downstream caspase pathways to trigger apoptosis. Although the membrane proximal components of TNF-α signaling are relatively well defined, the downstream mechanisms for triggering cellular behaviors depend on the wiring of the signaling network, which is strongly dependent upon cellular context. Because TNF-α activates a combinatorial complement of pathways that overlaps with those activated by other factors in the cellular environment, and because different cell types exhibit distinct responses to TNF-α, it is a challenge to decipher how TNF-α controls phenotypic outcomes in a physiological setting.

Dysregulation of TNF-α signaling contributes to several distinct chronic inflammatory conditions in the gut, such as inflammatory bowel disease, celiac disease, and peptic ulcer disease [5]–[7]. These conditions are characterized by alterations in intestinal homeostasis, which depends upon regulated interaction between the immune system, the intestinal epithelium, and the commensal flora residing in the intestinal lumen. Under homeostatic conditions, the unilaminar epithelium provides a barrier between the commensal flora and the immune system. As such, the epithelium is an active player, along with macrophages, dendritic cells, and neutrophils, in innate immunity within the intestine. When the barrier is intact, regulatory T (Treg) cells suppress adaptive immune responses. Loss of barrier function leads to activation of the adaptive immune component of the gut, resulting in inflammation. Different subtypes of T helper (Th) cells are activated in different types of chronic intestinal inflammation. For example, Th1/Th17 responses drive Crohn's disease, while Th2-like responses drive ulcerative colitis [8]. Recent studies have shown that inflammatory cytokines, such as those that define Th subsets, can also be produced by innate immune cells in the gut. Th17-like responses can arise from innate lymphoid cells (ILCs) [9]–[11] and Th-2-like responses from natural killer (NK) cells [12],[13].

Under both homeostatic and pathologic conditions, the activity of the innate and adaptive components of the intestine can strongly influence the behavior of the intestinal epithelium and vice versa. For example, T cell receptor (TCR)-γδ+ lymphocytes regulate the proliferation and differentiation of epithelial cells through extracellular factors such as keratinocyte growth factor-1 [13],[14]. T cells also direct the differentiation of epithelial cells by regulating Notch signaling, an important pathway in specifying the goblet cell lineage, which generates the mucus barrier [15]. Reciprocally, goblet cells can deliver antigens to dendritic cells to trigger T cell responses [16]. The heterologous cellular interactions that occur within the intestinal microenvironment create a situation where epithelial cells must receive and integrate mixtures of signals to dictate behaviors. The epithelial response to TNF-α takes place within this complex milieu, but the exact manner in which the environmental context modulates the integration of inflammatory signals to alter TNF-α-induced phenotypes in the intact tissue is not well understood.

Applying a systems biology perspective to explore communication mechanisms within the complex intestinal microenvironment will undoubtedly lead to novel insights into TNF-α biology, inflammatory diseases, and, more generally, heterologous cell interactions that occur as part of biological phenomena in complex tissues. Previously, we have demonstrated that the complex signaling effects elicited by TNF-α in the intestinal epithelium can be mathematically integrated into a signaling network state to predict epithelial cell behavior [17]. Here, we demonstrate that intestinal immune cells play a critical role in regulating the response of the epithelium to TNF-α. The effect is mediated by the pro-survival activity of MCP-1 and the pro-death activity of IFN-γ, two cytokines that, like TNF-α, exert pleiotropic effects on many cell types.

## Results

### Loss of Intestinal Lymphocytes, But Not the Commensal Flora, Alters the Epithelial Response to TNF-α

**Figure 1:**
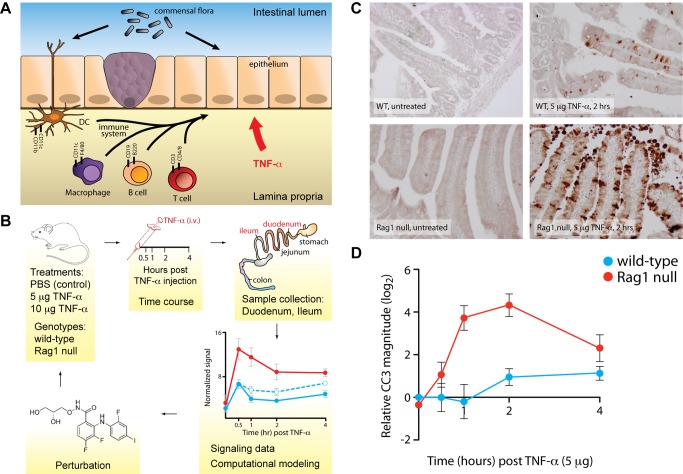
Loss of intestinal lymphocytes affects TNF-α-induced cell death in the intestinal epithelium. (A) Schematic representation of our driving hypothesis. Intestinal epithelial homeostasis is regulated by signals from the intestinal microenvironment. By extension, we hypothesized that perturbation of the commensal flora or components of the intestinal immune system (dendritic cells, B cell, T cells, macrophages) will affect how the epithelium responds to acute challenge with TNF-α. (B) Schematic of the experimental design. Wild-type or Rag1 null animals were exposed to TNF-α (5 or 10 µg in PBS) by intravenous injection. Mice were sacrificed at specific time points to compile a time course of TNF-α action. Protein lysates were derived from the duodena and ilea and then subjected to various analyses in order to collect datasets for mathematical modeling. Hypotheses based on computational models were tested by making perturbations and then the model was refined. (C) Immunohistochemistry for cleaved caspase 3 (CC3) in the duodena of untreated and treated animals. TNF-α-induced apoptosis occurs much more frequently in Rag1 null animals than in wild-type. (D) Quantification of caspase 3 cleavage by quantitative Western blotting, demonstrating the effect of Rag1 mutation on the timing and magnitude of TNF-a-induced apoptosis. CC3 data are normalized to vehicle-treated control and plotted on a log2 scale. Error bars represent the SEM for three mice.

We have previously used systems biology to characterize how cell intrinsic signaling regulates the response of the intestinal epithelium to TNF-α [17]. Because homeostasis within the epithelium is affected by components of the intestinal microenvironment, we hypothesized that non-cell-autonomous signaling might also play a significant role in the epithelial response to TNF-α (Figure 1A). To test how one component of the intestinal microenvironment—the immune component—might regulate the epithelial response to TNF-α, we compared responses of wild-type (WT) and recombinase-activating gene 1 (Rag1) knockout mice, which lack adaptive lymphocytes, to acute TNF-α exposure. We intravenously administered TNF-α (5 µg) to isogenic WT and Rag1 null animals and, over a time course of 4 h, measured the onset of apoptosis in the intestinal epithelium (Figure 1B). The duodena of Rag1 null mice displayed a qualitatively enhanced apoptotic response to TNF-α when compared to WT (Figure 1C). Consistent with our previous observations [17], exogenous TNF-α did not trigger apoptosis in the ilea of WT or Rag1 mutant animals (unpublished data). Careful quantification by Western blotting for cleaved caspase 3 (CC3, Figure S1A) revealed that the apoptotic response in Rag1 null mice differed from WT both in timing and in magnitude. TNF-α-induced apoptosis occurred earlier in Rag1 null mice than in WT and with increased magnitude (Figure 1D). These results demonstrate that the immune component of the intestine is critical for modulating the epithelial response to TNF-α in a non-cell-autonomous manner.

Mutation of Rag1 affects the development of both B and T cells. To determine whether loss of either of these types of lymphocytes is specifically responsible for the enhanced apoptotic response of the intestinal epithelium, we acutely treated animals that lacked either T cells or B cells with TNF-α. Interestingly, T cell receptor beta/delta (Tcrb/Tcrd) double null animals, which lack T cells, and Immunoglobulin heavy chain mu (Ighm) null animals, which lack B cells, both exhibited an enhanced apoptotic response to TNF-α relative to WT controls (Figure S1B), suggesting that both of the major immune cell types in the gut are required for dampening the epithelial TNF-α response.

While WT and Rag1 null mice differ in the presence of resident T cells within the intestinal lamina propria, we noted that acute treatment of WT mice with TNF-α also led to recruitment of additional T cells to the intestine (Figure S1C). To determine whether these recruited T cells are required to suppress TNF-α-induced epithelial apoptosis, we blocked their recruitment to the intestinal lamina propria from the mesenteric lymph nodes by pre-treating WT mice with a neutralizing antibody for mucosal addressin cell adhesion molecule-1 (MAdCAM-1), the endothelial ligand in the intestine for homing α4β7 integrins on T cells [18],[19]. Blocking MadCAM-1 abolished the recruitment of T cells to the lamina propria by TNF-α, but, as expected, did not significantly affect the presence of resident T cells (Figure S1C). Moreover, this perturbation did not alter TNF-α-induced apoptosis in intestinal epithelial cells (Figure S1D), indicating that recruited T lymphocytes are not likely to be important for modulating the epithelial apoptotic response.

The commensal flora constitutes another feature of the intestinal microenvironment that plays an important role in regulating epithelial homeostasis. As such, we sought to determine whether acute elimination of the endogenous microbiota would affect the response of the intestinal epithelium to TNF-α. Treatment of WT mice with broad-spectrum antibiotics eliminated the commensal flora population that could be cultured from fecal pellets (Figure S2A), but did not significantly affect TNF-α-induced apoptosis of intestinal epithelial cells (Figure S2B). Altogether, these results suggest that resident lymphocytes, but not the endogenous microbiota, play a major role in the short-term response of the intestinal epithelium to TNF-α-induced epithelial damage. It remains unclear, however, whether long-term depletion of gut microbes or antibiotic-resistant microbiota may play a role in the acute response to TNF-α.

### Mathematical Modeling Identifies an Epithelial Signaling Network That Distinguishes Apoptotic Phenotypes

**Figure 2:**
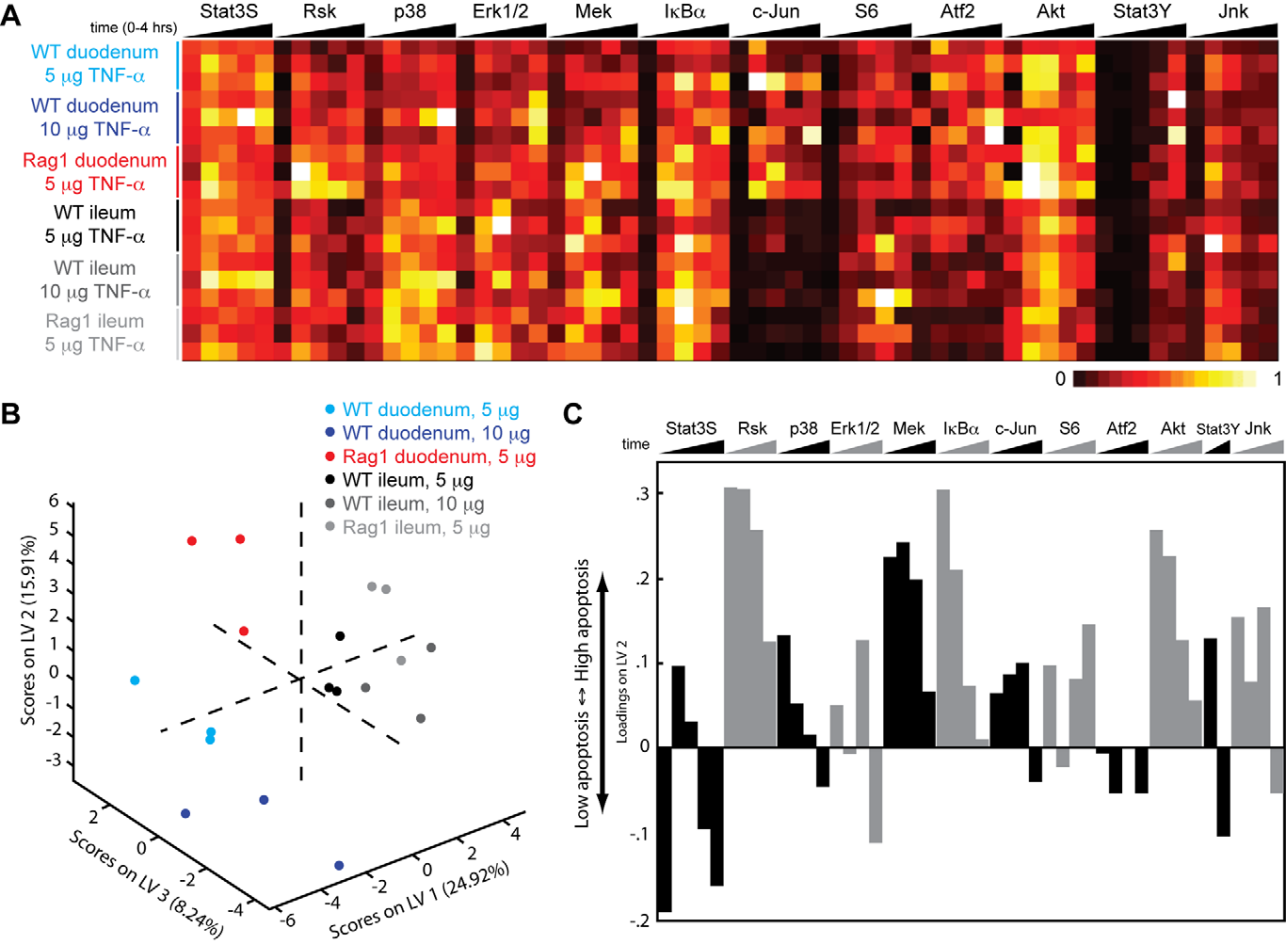
The epithelial TNF-α signaling network is altered by the absence of lymphocytes. (A) Measurement of protein phosphorylation in intestinal tissue lysates by Bio-Plex. The heat map is organized such that the columns represent the phospho-signal time courses, while the rows represent the experimental conditions. Different doses, regions, and genotypes are color-coded. Each phospho-signal is arranged sequentially as a function of time (left to right, 0 to 4 h). The intensity of the heat map represents the average of technical duplicates of the median fluorescence intensity (normalized to the highest value of each signal) resulting from each assay. Data are compiled from three independent experiments for each condition. (B) A three-dimensional PLSDA model constructed using signaling time courses obtained from the different conditions and their correlating apoptotic phenotypes. Each data point represents scores generated by the model, composed of all signaling time courses of a particular experimental condition mapped onto the three-dimensional latent variable space. The three orthogonal latent variable axes separate the scores by apoptotic phenotype: LV1 by the presence or absence of apoptosis, LV2 by the magnitude of apoptosis, and LV3 by the timing of apoptosis. The percentages on the axes represent the percent variance in the dataset captured by a particular LV. (C) Loadings on LV2, the latent variable that correlates the magnitude of TNF-α-induced apoptosis. The *y*-axis quantifies the positive or negative contribution of a particular signal to LV2.

To identify the mechanisms by which immune cells modulate the magnitude of TNF-α-induced apoptosis in epithelial cells, we first asked whether network-level signaling responses to TNF-α varied in the different genetic backgrounds. To sample the network state, we collected a time course dataset across central signaling pathways (Figure S3) from intestinal lysates using Bio-Plex signaling assays under various conditions: (i) late, low-magnitude apoptosis in the duodena of WT mice treated with 5 µg of TNF-α; (ii) early, low-magnitude apoptosis in the duodena of WT mice treated with 10 µg of TNF-α; (iii) early, high-magnitude apoptosis in the duodena of Rag1 null mice treated with 5 µg of TNF-α; and (iv) no apoptosis in the ilea of all animals, regardless of genotype or treatment (Figure 2A). Altogether, we collected 2,160 data points (45 animals, 2 sections per animal, 12 signals per sample, 2 technical duplicates) for this initial model-generating experiment. In addition to the expected biological variation observed between animals, we observed strong condition-induced variation (Figure S4). We then used our signaling dataset to construct a PLSDA model that accounts for the large increase in the magnitude of apoptosis seen in Rag1 null animals (Figure 2B). We selected a three-dimensional model based on the lowest classification error of calibration coupled to the lowest classification error of validation of the three duodenal classes (Figure S5A–C). The distinction between ileal samples by the model was suboptimal (Figure S5C), which was expected due to the similarity in phenotype (i.e., no apoptosis) and signaling of ileal samples under all conditions. It can be appreciated that the high-magnitude apoptotic phenotype is not just an extension of the early timing phenotype, but is in fact a clearly separate class, since these two phenotypic classes (timing and magnitude) lie on different axes (Figure 2B). Consequently, different mechanisms of signaling classify duodenal phenotypes based on timing or magnitude. Late activation of Erk, Mek, Rsk, p38, S6, and IκBα separates apoptotic phenotypes by timing (early from late), similar to our previous study (Figure S5D) [17]. By contrast, early signaling through Rsk, Mek, Akt, and IκBα distinguishes the apoptotic phenotypes by magnitude (Figure 2C). These observations indicate that magnitude and timing of apoptosis within the intestinal epithelium are controlled by distinct “axes” of signaling, suggesting that TNF-α-induced apoptosis is dysregulated in multiple ways in animals lacking adaptive lymphocytes.

### Computational Modeling of Cytokine Signaling Reveals a Non-Cell-Autonomous Network Controlling Apoptosis in the Intestinal Epithelium

**Figure 3:**
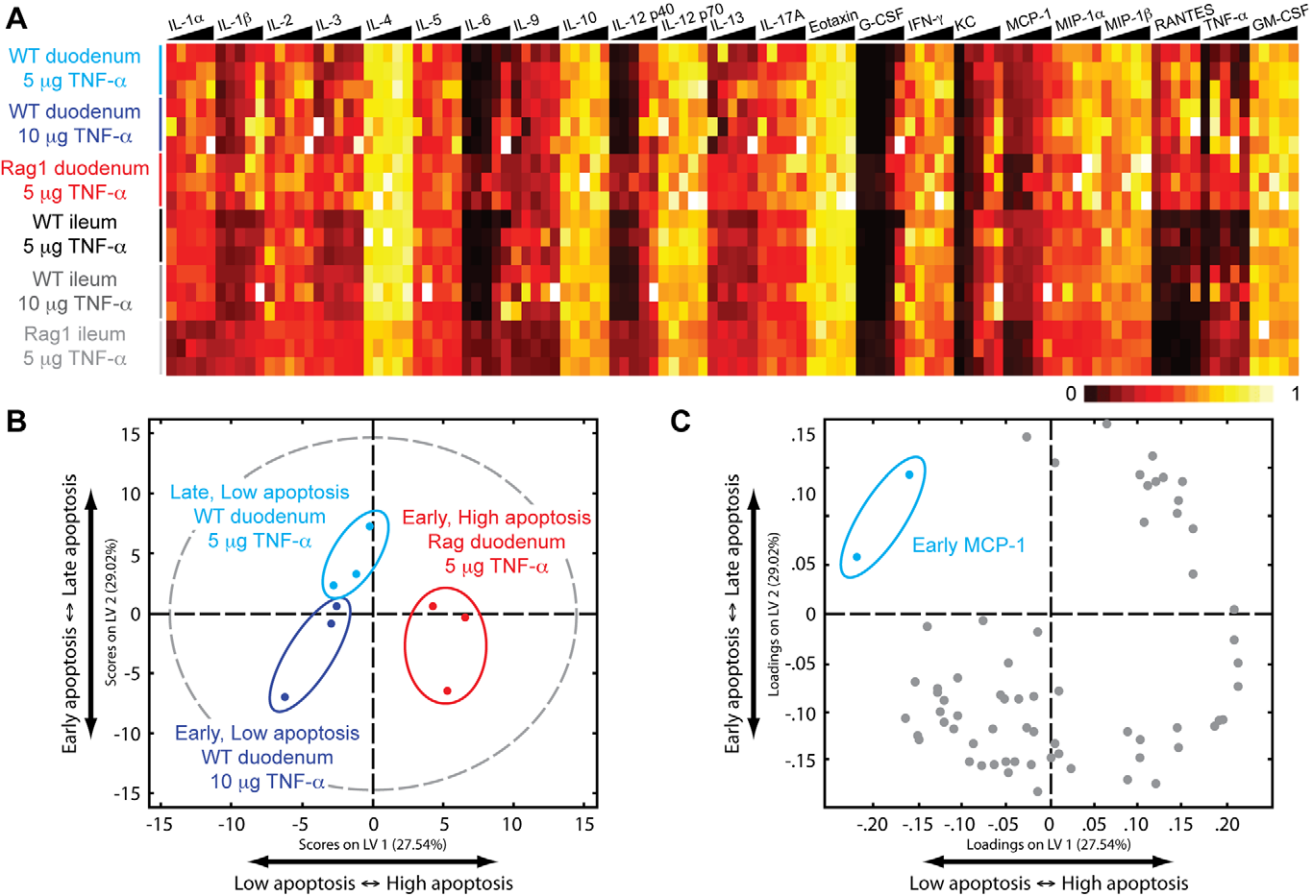
The cytokine milieu is modulated by the presence or absence of lymphocytes. (A) Measurement of cytokine protein levels in intestinal tissue lysates by Bio-Plex. The heat map is organized in a similar manner as Figure 2A. Data are compiled from three independent experiments for each condition. (B) A two-dimensional PLSDA model constructed using early cytokine data (0, 0.5, 1 h) obtained from the different conditions and their correlating apoptotic phenotypes. Each data point represents scores generated by the model, composed of the early time points of all cytokines measured of a particular experimental condition mapped onto the two-dimensional latent variable space. The dotted gray line represents the 95% confidence limit of the distribution of the scores. The two orthogonal latent variable axes separate the scores by apoptotic phenotype: LV1 by the magnitude of apoptosis and LV2 by the timing of apoptosis. The percentages on the axes represent the percent variance in the dataset captured by a particular LV. (C) Loadings for the PLSDA model plotted on LV1 and LV2. MCP-1 clusters most significantly with the lowest degree of apoptotic phenotype (low magnitude, late timing).

The observation that lymphocyte perturbation resulted in altered epithelial signaling and TNF-α response prompted us to investigate whether immune cells communicate with epithelial cells through cytokine networks. To examine whether cytokine expression is disrupted at a network level, we measured a panel of inflammatory cytokines in the intestine after TNF-α stimulation (4,140 data points—45 animals, 2 sections per animal, 23 signals per sample, 2 technical duplicates) (Figure 3A). The cytokine dataset contained a similar degree of variation as did the phospho-protein signaling dataset (Figure S6). We surmised that, with respect to modulation of TNF-α-induced apoptosis, the cytokine network lies upstream of the epithelial signaling network, and thus, late apoptosis can only be modulated by early alterations in cytokines. To identify cytokines that are important in modulating the apoptotic phenotype, we constructed a two-dimensional PLSDA model separating the three phenotypic classes (late/low apoptosis, early/low apoptosis, and early/high apoptosis) by early cytokine expression within the duodenum (Figure 3B). A variety of inflammatory cytokines were positively correlated with the high magnitude of TNF-α-induced apoptosis in Rag1 null duodenum, but only one, MCP-1 (monocyte chemotactic protein 1), correlated with the late and low magnitude of apoptosis in WT duodenum (Figure 3C), suggesting that MCP-1 is protective against TNF-α-induced apoptosis in the intestinal epithelium.

### MCP-1 Protects the Intestinal Epithelium from TNF-α-Induced Apoptosis

**Figure 4:**
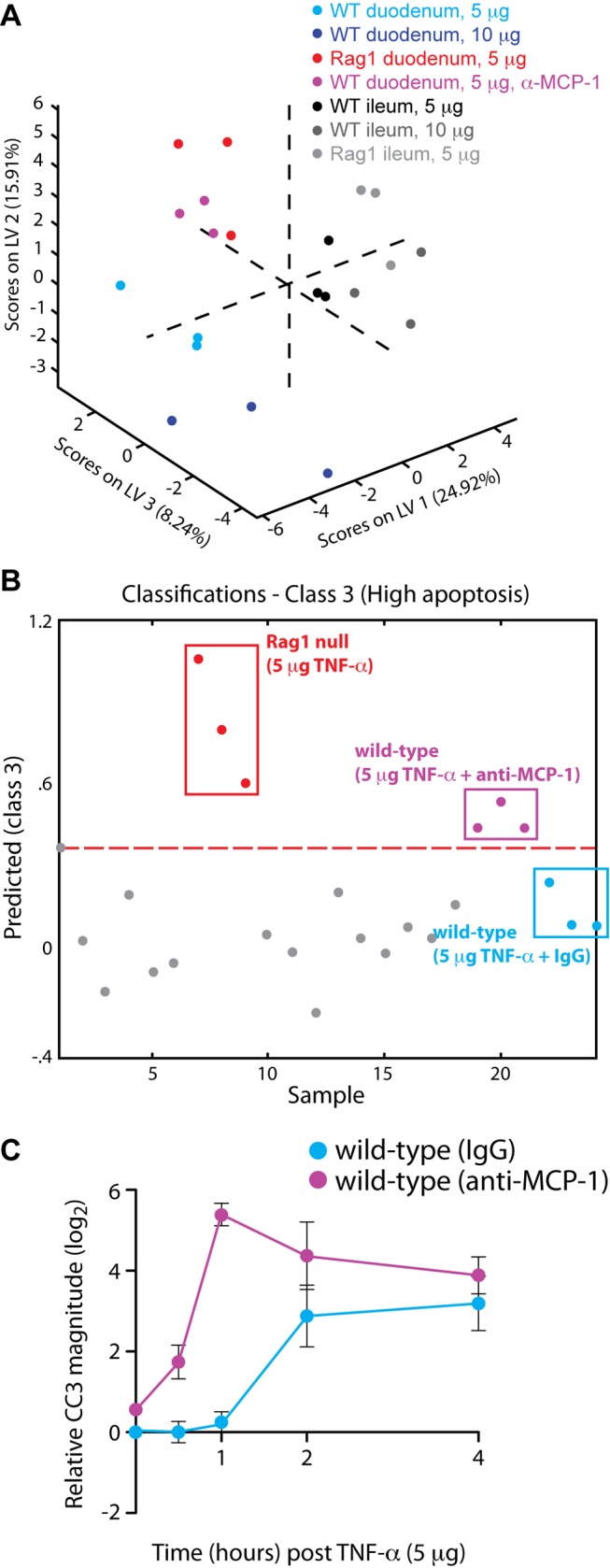
Neutralization of MCP-1 affects epithelial signaling and TNF-α-induced apoptosis in the intestine. (A) Effect of MCP-1 neutralization on TNF-α-induced signaling. Phospho-signaling data from the duodena of wild-type mice pre-treated with MCP-1 neutralizing antibody (magenta) were mapped onto the three-dimensional LV space to visually demonstrate the classification of these data with the original Rag1 null dataset. (B) Numerical classification of the wild-type anti-MCP1 signaling dataset into the Rag1 null class. The *y*-axis shows the numerical result calculated with the PLSDA function of classification into the “high apoptosis” phenotypic class (class 3). The broken red line is the threshold defining classification. Signaling data from the duodenal tissues of control mice pretreated with non-specific antibody (cyan) do not surpass the threshold for classification into this class. (C) Experimental results validating model predictions. Time course of caspase 3 cleavage induced by TNF-α, as determined by quantitative Western blotting after pretreatment with MCP-1 neutralizing antibody (magenta) or non-specific antibody (cyan). CC3 data are normalized to vehicle-treated control and plotted on a log2 scale. Error bars represent the SEM for three mice.

To test directly whether it singularly regulates the epithelial apoptotic response to TNF-α, we functionally neutralized MCP-1 in WT animals with anti-MCP-1 antibody prior to TNF-α administration. Next, we collected a signaling dataset representing a time course of TNF-α stimulation (5 µg) coincident with MCP-1 neutralization (360 data points—15 mice, 12 signals, 2 technical duplicates). Application of this signaling dataset to the original three-dimensional PLSDA model of signaling (Figure 2B) demonstrated that MCP-1 neutralization shifted the signaling data from the late/low apoptosis class (WT duodenum, 5 µg TNF-α) towards the early/high-magnitude apoptosis class (Rag1 null duodenum) (Figure 4A). Classification analysis revealed that the anti-MCP-1 signaling dataset classified solely into the high-magnitude apoptosis class (Figure 4B), but not into other classes (Figure S7). By contrast, the signaling data obtained from control WT mice pretreated with non-specific antibody (rat IgG) classified into the low-magnitude and late apoptosis classes, but not into the high-magnitude class (Figures 4B, S7). Our PLSDA model indicated that acute neutralization of MCP-1 primes the signaling network of intestinal epithelial cells from WT mice to respond to TNF-α in a similar manner as epithelial cells from Rag1 null mice. Consequently, we predicted that neutralization of MCP-1 in WT mice would lead to early and high-magnitude apoptosis after treatment with TNF-α. In order to validate this model-derived hypothesis, we measured TNF-α-induced apoptosis in the duodenum of WT mice pretreated with anti-MCP-1. Compared to mice pretreated with non-specific antibody, intestinal epithelial cells from mice treated with anti-MCP-1 underwent earlier and higher magnitude apoptosis (Figure 4C). These observations suggest that MCP-1 strongly attenuates TNF-α-induced apoptosis in the intestinal epithelium by causing systems-level shifts within the epithelial signaling network.

### MCP-1 Is Expressed by Secretory Epithelial Cells

**Figure 5:**
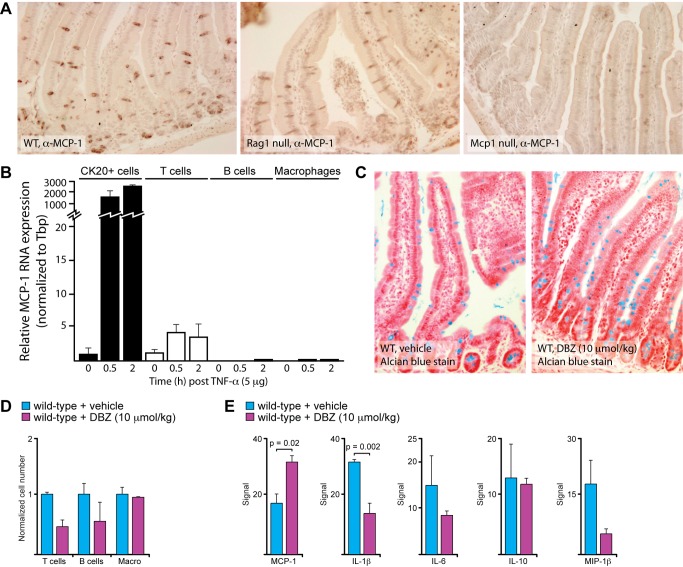
MCP-1 is expressed by secretory epithelial cells. (A) Expression of MCP-1 in the duodenum of wild-type, Rag1 null, and Mcp1 null mice as demonstrated by immunohistochemistry. The MCP-1-positive cells are goblet and Paneth cells. (B) Expression of MCP-1 in sorted cells. MCP-1 could not be detected in sorted B cells or macrophages. T cells express relatively low levels of MCP-1 in the basal state and induce MCP-1 5-fold after exposure to TNF-α. Epithelial cells also express relatively low levels of MCP-1 in the basal state and induce MCP-1 2,000-fold after exposure to TNF-α. (C) Increase in goblet cell number after treatment with DBZ. Intestinal tissue sections were stained with Alcian blue to highlight goblet cells. (D) Decrease in intestinal lymphocytes after treatment with DBZ. Immune cell types were quantified by FACS. (E) Expression of cytokines after treatment with DBZ. MCP-1 expression is increased in the intestines of animals treated with DBZ. Expression of IL-1β, IL-6, and MIP-1β decreased in animals treated with DBZ.

MCP-1, like TNF-α, is a pleiotropic cytokine that can affect a diverse array of cell types in a variety of ways. As such, it was unclear exactly how MCP-1 was functioning within the intestine to regulate the epithelial response to TNF-α. We set out to test multiple hypotheses relating to the relationship between MCP-1 and TNF-α-induced epithelial apoptosis. Based on an abundance of published work, our initial hypothesis was that lymphocytes secrete MCP-1 to recruit macrophages to the lamina propria and that this protects against TNF-α-induced epithelial apoptosis. Based on this hypothesis, we predicted that MCP-1 expression in the intestine would be detectable in intravillus lymphocytes. Surprisingly, we found that immunohistochemistry (IHC) for MCP-1 detected expression primarily in secretory intestinal epithelial cells, namely in goblet cells in the villi and Paneth cells at the base of the crypt (Figure 5A). This observation was consistent with epithelial cells being the primary source of MCP-1 in the intestine, but it did not rule out the possibility that lymphocytes express high levels of MCP-1 following stimulation with TNF-α. To address this issue, we isolated epithelial and immune cells from WT intestine before and after TNF-α treatment and performed real-time PCR (RT-PCR) for MCP-1. Among the immune cell types analyzed, we found that T cells, but not B cells or macrophages, expressed MCP-1 and that its expression was stimulated 4–5-fold by TNF-α (Figure 5B). Epithelial cells also expressed low levels of MCP-1 prior to exposure to TNF-α, but its expression was stimulated 2,000-fold by TNF-α (Figure 5B). This stimulation of expression is likely to be a significant underestimate of MCP-1 expression by secretory cells, as they comprise only about 10% of the epithelial cell population in the duodenum.

While these IHC and RT-PCR studies support the hypothesis that MCP-1 detected in the intestine is primarily derived from epithelial cells, a more direct way to link MCP-1 to secretory cells is to modify the number of those cells within the epithelium. Treatment of mice with dibenzazepine (DBZ), an inhibitor of γ-secretase, has been shown to increase the number of secretory epithelial cells in the intestinal epithelium [20]. We found that DBZ treatment increased the number of secretory epithelial cells and, at the same time, decreased the number of lymphocytes in the intestine (Figure 5C,D). The expression of MCP-1 was positively correlated with the number of secretory epithelial cells, with higher expression in animals treated with DBZ (Figure 5E). By contrast, most of the other cytokines that we measured in this experiment positively correlated with the number of lymphocytes, with lower expression in animals treated with DBZ (Figure 5E). Altogether, these studies are consistent with secretory epithelial cells providing the MCP-1 that protects the intestinal epithelium from TNF-α-induced apoptosis.

### MCP-1 Regulates Plasmacytoid Dendritic Cell Migration to the Intestine

While our data suggested that MCP-1 comes from epithelial cells, the mechanism of action of MCP-1 remained a question. Published studies have shown that this cytokine can act directly on epithelial cells, which also express the MCP-1 receptor, CCR2 [21]. Autocrine feedback loops have also been shown to protect intestinal epithelial cells from TNF-α-induced apoptosis using in vitro systems [22]. Based on these observations, we explored the hypothesis that MCP-1 functions in an autocrine manner to protect the epithelium against TNF-α-induced apoptosis. The major prediction of this hypothesis is that exogenous MCP-1 will protect the duodena of Rag1 null animals from TNF-α-induced apoptosis. Nevertheless, intravenous injection of Rag1 null animals with MCP-1 prior to TNF-α treatment failed to inhibit apoptosis, even though the tissue levels of MCP-1 were returned to that of WT (Figure S8A,B).

**Figure 6:**
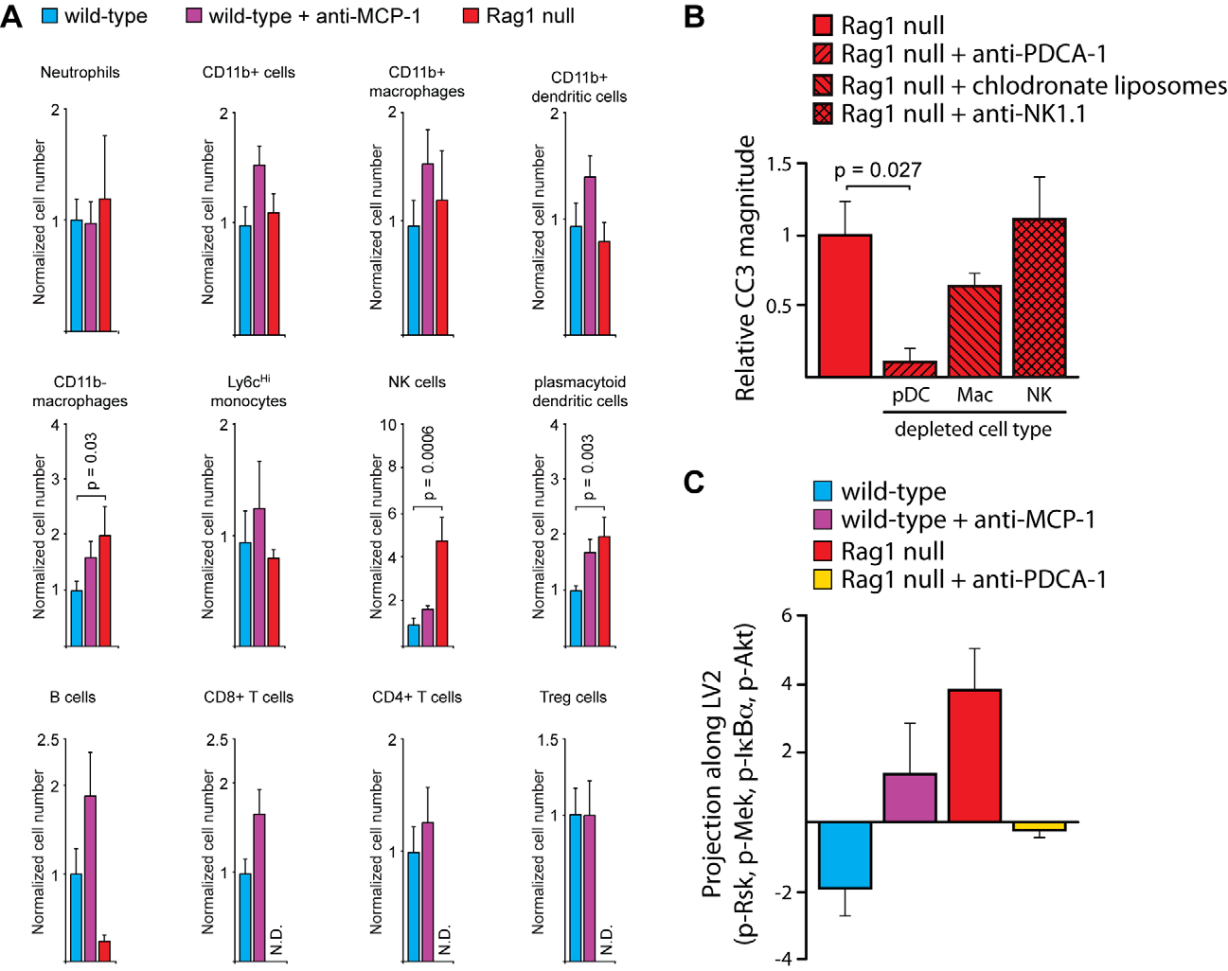
MCP-1 regulates the presence of plasmacytoid dendritic cells in the intestine. (A) Quantification of basal immune cell numbers by FACS. Data are normalized to the number of cells in the duodena of wild-type mice. Error bars represent the SEM for six animals. N.D., not detected. (B) Quantification of caspase 3 cleavage in the duodena of Rag1 null animals in which specific immune cell populations have been depleted. Data are normalized to Rag1 null control mice and error bars represent the SEM for three animals. (C) The projection of different perturbation conditions (wild-type, wild-type+anti-MCP-1, Rag1 null, and Rag1 null+anti-PDCA1) along LV2 based on p-Mek, p-IκBα, p-Rsk, and p-Akt were measured at 0.5 h post-TNF-α treatment. These phospho-signals were identified as the primary loadings for LV2, which classifies the apoptotic phenotype by magnitude, of the PLSDA model of epithelial phospho-signaling.

MCP-1 is mainly known as a chemo-attractant that recruits monocytes, macrophages, dendritic cells, and T cells to sites of inflammation. We next tested the hypothesis that epithelial cells secrete MCP-1 to recruit a protective immune cell type (possibly macrophages) to the intestine. Nevertheless, by performing FACS analysis on immune cell populations from the gut, we found no evidence that Rag1 null animals or WT animals treated with anti-MCP-1 had fewer intestinal macrophages than control WT animals (Figure 6A).

All of our initial hypotheses relating to the function of MCP-1 were developed under the assumption that WT animals are actively resistant to TNF-α-induced apoptosis. Because our experimental results failed to support these hypotheses, we explored the possibility that Rag1 null mice are actively sensitive to TNF-α-induced apoptosis. We tested the hypothesis that the low levels of MCP-1 in Rag1 null intestines led to an increased number of deleterious immune cells relative to WT animals. Indeed, we identified three distinct immune populations that were over-represented in Rag1 null intestines: CD11b- macrophages, natural killer (NK) cells, and plasmacytoid dendritic cells (pDCs) (Figure 6A). To determine the functional significance of these specific cell types, we ablated them in Rag1 null animals (Figure S8C,D). Ablation of macrophages and NK cells had no significant effect on TNF-α-induced apoptosis in Rag1 null mice, but depletion of pDCs was strongly protective (Figure 6B). Consistent with the notion that pDCs potentiate TNF-α-induced apoptosis, treatment of WT mice with anti-MCP-1 led to an increase of intestinal pDCs coincident with increased TNF-α-induced apoptosis (Figure 6A).

### pDCs Regulate the Epithelial Signaling Network by Increasing Tissue Levels of IFN-γ

Our data implicate pDCs in actively sensitizing the intestinal epithelium to TNF-α-induced apoptosis. We next investigated whether the increased or decreased presence of pDCs is sensed through the epithelial signaling network to change the magnitude of TNF-α-induced apoptosis. Our original modeling analysis comparing WT and Rag1 null animals identified four early signals (0.5 h post-TNF-α) that combinatorially contribute to the magnitude of TNF-α-induced apoptosis: p-Rsk, p-Mek, p-IκBα, and p-Akt (Figure 2C). We measured these four signals after perturbations that increase (anti-MCP-1 in WT mice) or decrease (anti-PDCA-1 in Rag1 null mice) pDCs and examined how the signals from these conditions project onto LV2 (which separates low- and high-magnitude apoptosis) of the PLSDA model based on the four signals. Increasing intestinal pDCs in WT mice reversed the direction of projection by these signals onto LV2, signifying that the behavior of the epithelial signaling network is modified in such a way that gives rise to increased apoptosis (Figures 6C, S9). Alternately, decreasing pDCs in Rag1 null mice reversed the projection, consistent with the experimental observation that ablation of pDCs decreased the magnitude of TNF-α-induced apoptosis in these mice (Figures 6C, S9). Based on these studies, we conclude that changes in the composition of intestinal immune cell populations remodel the signaling network in epithelial cells, ultimately leading to an altered response to TNF-α.

**Figure 7:**
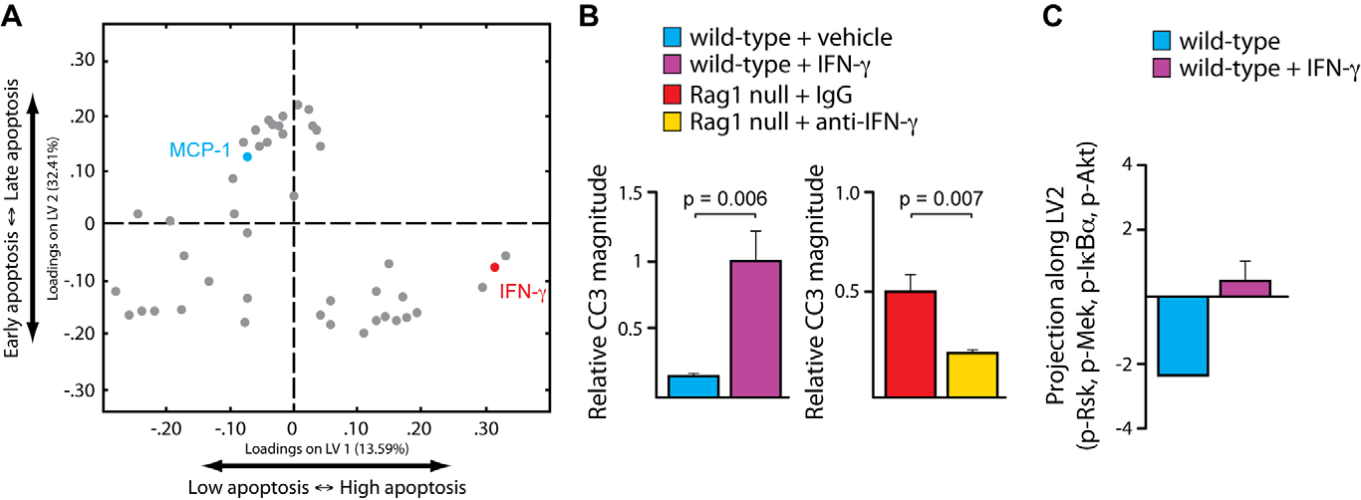
IFN-γ regulates TNF-α signaling and apoptosis in a pDC-dependent manner. (A) A two-dimensional PLSDA model constructed using early cytokine data from four different experimental conditions (wild-type+IgG, wild-type+anti-MCP-1, Rag1 null+IgG, Rag1 null+anti-PDCA-1) in duodenal lysates. The loadings plot for this four-condition PLSDA model identifies IFN-γ as correlating with high-magnitude apoptosis. (B) Caspase 3 cleavage induced 2 h post-TNF-α administration in the duodena of wild-type mice co-treated with PBS (cyan) or IFN-γ (purple) and Rag1 null mice pretreated with IgG (red) or anti-IFN-γ for 2 h (yellow). Error bars represent SEM for three mice. (C) Projection of different perturbation conditions (wild-type and wild-type+IFN-γ) along LV2 based on p-Mek, p-IκBα, p-Rsk, and p-Akt measured at 0.5 h post-TNF-α treatment.

The major question that remained was: How does the change in the number of pDCs affect the epithelial signaling network? We posited that pDCs might communicate with epithelial cells, directly or indirectly, using cytokines. Based on our original PLSDA model, we identified MCP-1 as being protective because it correlated with late/low apoptosis (Figure 3C). We speculated that a similar analysis might allow us to identify cytokines that correlated with early/high apoptosis (i.e., those that might be functioning downstream of pDCs). Nevertheless, the right side of the model, which correlates to high-magnitude apoptosis, is obscured by the multitude of signals present (Figure 3C). This result could arise from one of two scenarios: (1) many signals combinatorially contribute to high-magnitude apoptosis, or (2) many of the signals are just correlative and not causative, due to the lack of a sufficient number of perturbations to constrain the model. To gain a better perspective of the cytokines that modulate the magnitude of apoptosis, we augmented our model with early cytokine data from our four distinct experimental conditions (WT, WT treated with anti-MCP-1, Rag1 null, and Rag1 null treated with anti-PDCA-1) (Figure S10). The resulting modified PLSDA model identified high interferon gamma (IFN-γ) levels as correlating with a high magnitude of TNF-α-induced apoptosis (Figure 7A).

To test whether IFN-γ plays a mechanistic role in the potentiation of TNF-α-induced apoptosis by pDCs, we first treated WT animals with exogenous IFN-γ concurrent with TNF-α. We found that a single injection of IFN-γ was sufficient to enhance TNF-α-induced apoptosis in the WT intestinal epithelium (Figure 7B). Consistent with this observation, neutralization of IFN-γ in Rag1 null animals was strongly protective (Figure 7B). Similar to neutralization of MCP-1, treatment with ectopic IFN-γ reversed the direction of signal projection onto LV2 of our PLSDA model, suggesting that it influences the epithelial signaling network downstream of pDCs (Figure 7C).

## Discussion

**Figure 8:**
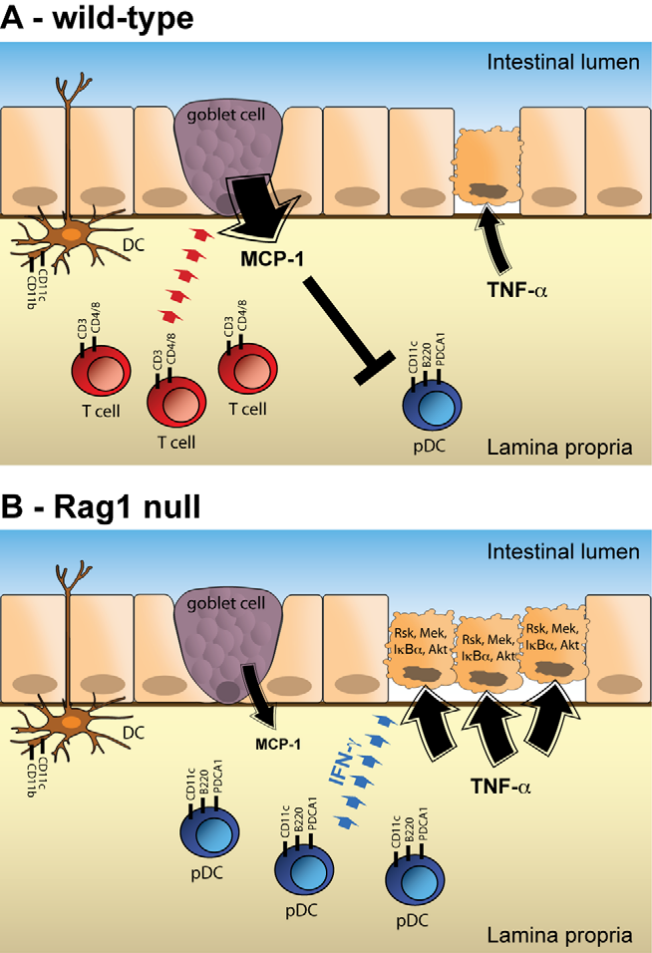
A model for regulation of TNF-α-induced apoptosis in the intestine. (A) Regulation of TNF-α response in the wild-type duodenum. In this context, resident T-cells promote MCP-1 expression by epithelial goblet cells. This MCP-1 functions to restrict pDC recruitment to the lamina propria. In the absence of pDCs, apoptosis induced by exogenous TNF-α is relatively low. Neutralization of MCP-1 in wild-type animals results in recruitment of pDCs and enhanced TNF-α-induced apoptosis. (B) Regulation of TNF-α response in the Rag1 null duodenum. The lack of intestinal T cells in Rag1 mice leads to a reduction in MCP-1 expression by goblet cells, allowing for recruitment of pDCs to the lamina propria. These pDCs influence the activation state of TNF-α-induced signaling pathways in epithelial cells, ultimately resulting in enhancement of TNF-α-induced apoptosis. Ablation of pDCs in Rag1 null mice restores the signaling network, and therefore the response to TNF-α, to a wild-type state. (Note, the cell surface markers used to identify each of the relevant immune cell types is specified.)

Tissue homeostasis is maintained by a complex biological network consisting of cell intrinsic and extrinsic factors. Data-driven computational modeling provides a powerful framework for understanding how complex networks are established and maintained in a heterogeneous system [23]. The mammalian intestine is a prime example of a complex, heterogeneous system in which homeostasis is controlled by interactions between the intestinal epithelium, the flora residing within the gut lumen, and the immune system residing in the lamina propria. To begin to understand how signaling within this complex system is regulated to maintain homeostatic balance, we applied a top-down approach to identify, and then disrupt, components within the system that modulate cell death in response to the proximal inflammatory stimulus tumor necrosis factor alpha (TNF-α). We initially observed that Rag1 mutant animals exhibited a strongly enhanced apoptotic response to TNF-α relative to WT controls (Figure 1C,D). To understand the mechanisms underlying the phenotypic difference between WT and Rag1 null animals, we employed an in vivo systems biology approach that integrated immunological, cytokine, phospho-protein signaling, and phenotypic data. Our results demonstrated a critical link between plasmacytoid dendritic cells (pDCs) and the intestinal epithelial cell signaling network in modulating TNF-α-induced apoptosis, and that this interaction was regulated by monocyte chemotactic protein-1 (MCP-1) (Figure 8).

MCP-1 belongs to the CC chemokine group, whose members are characterized by two adjacent cysteine residues in their N-termini. MCP-1 is known to signal through the G protein coupled receptor CCR2 to induce a variety of chemo-attractant functions. CCR2 expression was initially established in monocyte and macrophage cell lines, and hence, most studies of MCP-1 function have focused on mononuclear cell recruitment. Nevertheless, CCR2 is also expressed by other immune and non-immune cell types [24]–[26]. As a result, MCP-1 can influence the behavior of many different types of cells. In the intestine, MCP-1 is thought to play a role in regulating homeostasis because it has been linked to intestinal inflammation [27]–[30]. The precise mechanisms linking MCP-1 to gut inflammation are not clear, however. In our study, MCP-1 expression negatively correlated with the magnitude of TNF-α-induced apoptosis in the intestinal epithelium (Figure 3C), which was accompanied by alterations in the epithelial TNF-α signaling network (Figure 2). Consequently, neutralization of MCP-1 in WT animals changed both the network-wide signaling response and the phenotypic response to TNF-α (Figure 4A,C). These results suggested that MCP-1 plays an important role in regulating intestinal epithelial homeostasis by altering the proximal sensitivity of the inflammatory signaling network of epithelial cells.

Our major goal in this study was to understand how heterologous interactions within the intestinal microenvironment play a role in regulating the epithelial response to TNF-α. Our initial hypothesis connecting MCP-1 to the epithelial TNF-α response was that it is expressed by lymphocytes, and that its presence affected epithelial sensitivity to TNF-α. Our results painted a more complex picture, however. For example, we found that MCP-1 was produced primarily by secretory epithelial cells (Figure 5). It is not yet clear how adaptive lymphocytes regulate MCP-1 expression by epithelial cells. One way for lymphocytes to communicate with goblet and Paneth cells would be through cytokines or chemokines. If such a mechanism were operating in this system, we would have expected to find a signal that positively correlated with MCP-1 in our PLSDA model, but we did not (Figure 3C). Our failure to find a co-correlated signal likely results from the limited scope of the cytokine analysis afforded by Bio-Plex analysis. By extension, measurement of a larger panel of signals may reveal the proximal regulator of MCP-1 expression by secretory epithelial cells.

Two pieces of evidence suggested that MCP-1 indirectly influences epithelial signaling by playing a proximal role in regulating the identity of the immune cell complexion of the intestinal lamina propria. First, the lack of lymphocytes in Rag1 null mice was associated with an enhanced presence of several other immune cell types (Figure 6A), which can be construed as an adaptation at the organismal level to the substantial perturbation of the immune system. We also found that neutralization of MCP-1 in WT mice broadly affected the presence of immune cell populations in the intestine (Figure 6A). While several immune cell types were affected, we determined experimentally that the increased presence of plasmacytoid dendritic cells (pDCs) sensitized epithelial cells to TNF-α-induced apoptosis and that MCP-1 acted upstream to regulate the number of pDCs in the intestine. Because MCP-1 is primarily known as a chemo-attractant, the mechanism underlying the chemo-repulsive activity of MCP-1 on pDCs, for example whether this is direct or indirect, is still unclear. One potentially interesting mechanism to consider is that innate lymphoid cells (ILCs), which are not affected by the Rag1 mutation, may play a role. ILCs play an important role in intestinal inflammation by producing pro-inflammatory cytokines, such as IL-17 and IFN-γ [31],[32]. Previous studies have shown that adaptive immune cells are important for suppressing the inflammatory activity of innate immune cells [33],[34]. Thus, the lack of adaptive lymphocytes in Rag1 null animals is likely associated with hyperactivity of resident ILCs. By extension, it is possible that ILCs may be mediating the recruitment of pDCs. Note, however, that although one population of ILC (NK cells) is increased in Rag1 null mice, depletion of these cells did not affect TNF-α-induced apoptosis (Figure 6B).

Unlike conventional dendritic cells, whose main function is to sense and present antigen, pDCs are a special class of dendritic cells that secrete cytokines. Although evidence supports the existence of a common progenitor for both types of dendritic cells, pDCs exhibit morphologies, expression profiles, and homing properties of secretory lymphocytes [35]. pDCs also express high levels of TLR7 and TLR9 to sense viral infections and mount type 1 interferon responses [36]. In addition to their role in infection, pDCs are thought to play a role in autoimmune diseases, such as lupus and psoriasis [37]. TNF-α also plays a central role in the pathology of autoimmune disease [38]. Our signaling data demonstrate that several TNF-α-dependent epithelial signaling pathways were hyper-activated in the context of increased numbers of pDCs. Surprisingly, although pDCs were associated with enhanced TNF-α-induced apoptosis, we found that these hyper-activated pathways were primarily known to be involved in mitogenesis and cell survival. While it is common for cells to induce pro-survival feedback loops following an apoptotic stimulus [22], the effect we have characterized in the intestinal epithelium in vivo is mostly likely due to proximal regulation of the signaling network because apoptosis occurs at a later time point (1 h post-TNF-α, Figure 1D) than signal activation (0.5 h post-TNF-α, Figure S4). It is not unprecedented for pathways that normally function to promote proliferation or survival to promote cell death under specific circumstances, however. For example, while MAPK signaling normally promotes proliferation, ERK is required for cisplatin-induced apoptosis in HeLa cells [39]. We speculate that in the intestinal epithelium, the combinatorial activation of Rsk, Mek, Iκβα, and Akt by TNF-α is interpreted as a strong pro-death signal, even though, individually, each of these pathways promotes survival. How do pDCs modulate the activation of these pathways by TNF-α? Computational modeling identified interferon gamma (IFN-γ) as one of the cytokines that positively correlated with high-magnitude apoptosis and, therefore, high numbers of pDCs (Figure 7A). Perturbation of IFN-γ in WT and Rag1 null animals shifted both the epithelial signaling networks and the apoptotic phenotypes (Figure 7), indicating that this cytokine functions downstream of pDCs to potentiate TNF-α-induced apoptosis in the intestinal epithelium. The connection between pDCs and IFN-γ was somewhat surprising, as pDCs are typically associated with type I interferon response, which includes IFN-α [40]. Consistent with our observations, however, pDCs have also been shown to secrete IFN-γ [41],[42].

In this study, we have uncovered a novel interaction network within the intestinal environment that controls the epithelial response to TNF-α (Figure 8). In this network, resident adaptive lymphocytes influence MCP-1 production by secretory epithelial cells, which affects recruitment of IFN-γ-expressing pDCs to the lamina propria, ultimately leading to modulation of the epithelial signaling network. Additional studies are required to determine whether other cell types within the intestinal microenvironment, and cytokines derived from those cell types, function within the network. These studies demonstrate that, in the complex in vivo environment, perturbation of a single compartment within the network does not affect phenotypic behaviors in a univariate manner, but instead causes systems-level shifts in multiple components that synergistically alter outcomes.

## Materials and Methods

### Animals, TNF-α Time Courses, and Sample Isolation

All of the experimental animals used in this study were 8-wk-old mice on a C57BL/6J genetic background. For TNF-α time courses, mice were anesthetized with Avertin (tribromoethanol, 250 mg/kg in 500 µl PBS, Sigma-Aldrich), and then administered 5 or 10 µg of recombinant mouse TNF-α (Abazyme) in phosphate-buffered saline (PBS, 50 µl total volume) by retro-orbital injection. Mice from the same set were treated at the same time, but were sacrificed at various time points. Control animals were anesthetized, injected with PBS (50 µl) by retro-orbital injection, and then sacrificed 2 h later.

For sample collection, animals were sacrificed and the small intestine was removed and washed in PBS supplemented with protease inhibitor. Duodenal and ileal tissues were lysed and homogenized immediately in Bio-Plex lysis buffer (Bio-Rad), or else fixed with formalin for subsequent immunohistochemical analysis. Duodenal samples were obtained from the 1 cm of small intestine immediately adjacent to the stomach. Ileal samples were obtained from the 3 cm of small intestine immediately adjacent to the cecum. All animal work performed in this study was undertaken according to approved protocols and animal welfare regulations as put forth by the Subcommittee on Research Animal Care (SRAC) at the Massachusetts General Hospital.

### Perturbations

For cytokine neutralization experiments, anti-MCP-1 antibody (2 mg/kg in 50 µl PBS, R&D Systems), anti-IFN-γ (2 mg/kg in 50 µl PBS, R&D Systems), or anti-MAdCAM1 (2 mg/kg in 50 µl PBS, BD Pharmingen) was administered by retro-orbital injection 2 h prior to the TNF-α time course. Exogenous MCP-1 (2.5 µg in 50 µl PBS, Abazyme) or IFN-γ (1 µg in 50 µl PBS, R&D Systems) was administered by retro-orbital injection concurrent with TNF-α treatment. To increase the number of secretory epithelial cells, animals were treated daily for 5 d with DBZ (10 µmol/kg, Santa Cruz Biotechnology) and then treated with TNF-α. DBZ was prepared as described previously [20].

To perturb the microbiota, mice were given drinking water supplemented with broad-spectrum antibiotics (200 µg/ml ampicillin, 50 µg/ml Primaxin, Merck) for 10 d prior to the TNF-α time course. To confirm the effect of the antibiotic, one fecal pellet was freshly collected from each animal, homogenized in Ringer's solution (Fisher), and plated on Rose/MacConkey agar plates (Hardy Diagnostics) immediately in a 1∶5 dilution.

To ablate immune cells, mice were administered anti-NK1.1 (12 mg/kg, eBiosciences PK136), anti-PDCA-1 (20 mg/kg, Miltenyi Biotec), or clodronate liposomes (250 µl, Encapsula) for 48 h prior to the TNF-α time course.

### Immunohistochemistry and Apoptotic Measurements

Standard immunohistochemistry procedures were followed. Slides were stained with anti-cleaved caspase 3 (Cell Signaling, 9661) with EDTA antigen retrieval. The immunohistochemistry procedure was altered for intracellular cytokine staining for MCP-1 such that all solutions were supplemented with 0.01% saponin (Sigma-Aldrich). Slides were stained with anti-MCP-1 (Abcam, ab8101, ECE.2) with citrate antigen retrieval. Apoptosis was quantified by quantitative Western blotting analysis of cleaved caspase 3 (CC3, Cell Signaling) in tissue lysates. Western blots were quantified on a LI-COR Odyssey infrared imaging system. Each measurement was normalized to CC3 values from a control TNF-α time course (wild-type mice) loaded on the same gel.

### Signaling and Cytokine Analyses

Quantitative measurements of protein phosphorylation were collected from lysed tissue samples using Bio-Plex (Bio-Rad), a multiplexed, bead-based system for analyzing total and phospho-protein levels in suspension [43]. The following signals were measured: phosphorylated inhibitor of nuclear factor B α (p-IκBα, Ser32, and Ser36), phosphorylated c-Jun N-terminal kinase (p-Jnk, Thr183, and Tyr185), p-Mek1 (Ser217 and Ser221), p-Erk1 (Thr202 and Tyr204) and p-Erk2 (Thr185 and Tyr187), p-Rsk (Thr359 and Ser363), p-p38 (Thr180 and Tyr182), p-c-Jun (Ser63), p-Atf2 (Thr71), p-Akt (Ser473), p-S6 (Ser235 and Ser236), phosphorylated signal transducer and activator of transduction 3 (p-Stat3, Ser727), p-Stat3 (Tyr705), and total Mek1. Tissue lysates were quantified via BCA (bicinchoninic acid; Pierce) and equal amounts of protein from each sample were used for the Bio-Plex assays: 4 µg of proteins was used for assays of p-Jnk, p-Akt, p-Stat3 (Tyr705), and p-Atf2, whereas 1.5 µg of proteins was used for the rest of the assays. These protein amounts were determined experimentally to be within the linear ranges of activity of these assays on small intestinal tissue samples before this analysis. Quantitative measurements of cytokine protein levels were collected from lysed tissue samples by Bio-Plex using Mouse Cytokine 23-plex kit: IL-1α, IL-1β, IL-2, IL-3, IL-4, IL-5, IL-6, IL-9, IL-10, IL-12 (p40), IL-12 (p70), IL-13, IL-17A, eotaxin, G-CSF, GM-CSF, IFN-γ, KC, MCP-1, MIP-1α, MIP-1β, RANTES, and TNF-α. Each Bio-Plex measurement was normalized to the corresponding measurements from a control TNF-α time course (wild-type mice) on the same plate.

### Quantitative Modeling

PLSDA modeling was done as described previously [17],[22],[44]. Statistical models were constructed with MATLAB PLS Toolbox 6.5 (Eigenvector), with each biological replicate treated as an individual sample so that the model could account for biological variability. The input matrix was organized such that all time points of the time courses for all signals were included for each individual sample. Data were normalized by mean centering and variance scaling. Cross-validation was performed with the contiguous block method.

### Fluorescence Activated Cell Sorting

After dissection, the 3 cm of duodenum of each mouse was collected and washed in PBS with 0.5% BSA and 2 mM EDTA. The intestine was weighed, homogenized mechanically, and incubated in 0.2 mg/mL collagenase in DMEM for 1 h at 37 degrees to separate the epithelial cells, prior to single cell suspension preparation with a cell strainer.

For FACS analysis of immune cells at a systems level, the single cell suspensions were divided into three aliquots for staining. The first aliquot was stained with fluorescently conjugated antibodies for CD90.2, NK-1.1, CD11b, F4/80, Ly-6c, CD103, B220, Ly-6G, CD49b, CD11c, and TER-119 for 1 h. The second aliquot was stained for B220, CD11c, NK-1.1, CD11b, CD49b, and PDCA1 for 1 h. The third aliquot was fixed with Cytofix/Cytoperm solution (BD Biosciences) and stained for CD3, CD8, and CD4 for 1 h, and washed and stained for Fox3p overnight. All antibodies were obtained from BD Biosciences. Stained samples were analyzed with a BD LSRII flow cytometer.

For experiments in which RNA was isolated from sorted cells, the preparation of the single cell suspension was altered such that the homogenized sample was incubated in 1 mg/mL collagenase in DMEM for 30 min at 37 degrees. For sorting epithelial cells, the sample was stained with an antibody for cytokeratin 20 (Acris). T cells were sorted with CD8 or CD4, B cells with CD19 or B220, and macrophages and dendritic cells with CD11C or F4/80. Sorting was performed with a FACSAria III (BD) directly into RLT buffer with β-mercaptoethanol. RNA was isolated from each population using an RNAeasy microkit (Qiagen).

### MCP-1 Expression Analysis

Real-time RT-PCR for MCP-1 was performed on the RNA samples using standard procedures on an ABI 7900HT (Applied Biosystems). The RNA expression level of each sample was compared to a standard sample on the plate, and normalized using TBP, HPRT, and GAPDH house-keeping genes to ensure the reproducibility of the results across different cell types, with the formula:
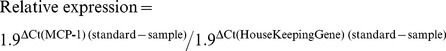
The above formula represents MCP-1 mRNA expression per unit of housekeeping gene expression. MCP-1 mRNA contribution for each cell population was calculated by multiplying the MCP-1 expression/unit by the number of cells in each population as determined by FACS analysis.

## Supporting Information

Figure S1 Effects of lymphocyte perturbation on acute TNF-α-induced apoptosis. (A) An example of a Western blot for caspase 3 cleavage and β-Tubulin in the duodena of Rag1 null mice (left) and wild-type mice (right) as a time course following TNF-α (5 µg) stimulation. (B) Caspase 3 cleavage induced 2 h post-TNF-α (5 µg) administration within the duodena of wild-type control mice (cyan), Rag1 null mice (red), TCRβ/δ (Tcrb/Tcrd) null mice (dark red), and immunoglobulin heavy chain mu (Ighm) null mice (brown). Data are normalized to wild-type control mice and error bars represent SEM for three mice. (C) Quantification of CD8+ T cells in the duodena of wild-type mice by FACS in wild-type controls (clear) or mice pretreated with anti-MadCAM1 (2 mg/kg) for 2 h (shaded). Treatment with anti-MadCAM1 prevents TNF-α-induced recruitment of T cells to the intestine. Error bars represent the SEM for three mice. (D) Caspase 3 cleavage within the duodena of wild-type control mice (white), Rag1 null mice (green), and wild-type mice pretreated with anti-MadCAM1 (shaded). Data are normalized to wild-type control mice and error bars represent the SEM for three mice.
(PDF)

Figure S2 Acute TNF-α-induced apoptosis in the intestine is not affected by modulation of the microbiota. (A) Fecal flora of wild-type mice on normal drinking water or on drinking water supplemented with broad spectrum antibiotics (200 µg/ml ampicillin, 50 µg/ml Primaxin) for 10 d. Fecal flora were plated on MacConkey agar (pink) and Rose agar (red). (B) Time course of caspase 3 cleavage induced by TNF-α as determined by quantitative Western blotting in the duodena of wild-type mice after antibiotic treatment above (broken line) or normal drinking water (solid line). Data are normalized to the peak signal of the wild-type control. Error bars represent the SEM for three mice.
(PDF)

Figure S3 Signaling network sampled in our analysis. Here, we present a manually curated network of inflammatory signaling according to canonical pathways. We depict components and connections that are included in our analysis (Input, TNF-α in yellow; Signals, cytokines in light blue, phospho-proteins in dark blue; Response, cleaved caspase 3 in red). Note that additional connections between the nodes likely exist, and note the presence or absence of particular connections that are cell context dependent.
(PDF)

Figure S4 Time courses of protein phosphorylation signals activated following exposure to TNF-α in vivo. Data points are the means of the median fluorescent intensities resulting from the phospho-protein assays, normalized to a loading control dataset on each plate. The left side of each graph set contains the duodenal data, while the right side contains the ileal data. The color scheme is based on genotype (cyan for wild-type, red for Rag1 null), while the solid and broken lines represent low (5 µg) and high (10 µg) doses of TNF-α, respectively. Error bars represent the SEM for three mice.
(PDF)

Figure S5 Modeling signaling network shifts in modulating TNF-α-induced apoptosis. (A) A 3-D PLSDA model describes dosage, regional, and genotypic effects on TNF-α-induced apoptosis. Error of classification into class 1 (late/low apoptosis, cyan), class 2 (early/low apoptosis, blue), and class 3 (early/high apoptosis, red) of calibration data, using models with increasing numbers of latent variables. The average error across all classes (black) includes the first three classes and the three ileum classes (no apoptosis). Classification error is defined as the probability of misclassifying a sample given the sample distribution and a model. (B) Error of classification into classes 1, 2, and 3 of data kept out of the cross-validation procedure, using models built with the rest of the data, with increasing numbers of latent variables. The average error across all classes (black) includes the first three classes and the three ileum classes (no apoptosis). Note that the average error is greater than the error for individual classes due to the fact that the ileum classes (no apoptosis) are very similar at the signaling and phenotypic level and cannot be distinguished from one another. (C) Receiver operating characteristic (ROC) curves depicting specificity (1-false positive rate) versus sensitivity (true positive rate) as the numerical threshold for classification is changed. The quality of a classification model can be evaluated by the degree by which the curves deviate from the center diagonal, with more deviation describing a better classification. ROC curves for the six classes using a PLSDA with three LVs. The red circle represents the discriminant threshold selected for a model. (D) Loadings on LV3, the latent variable for describing the timing of apoptosis. The *y*-axis quantifies the positive or negative contribution of a particular signal to LV3. Late signals correlate to an early apoptotic phenotype, consistent with our previous findings.
(PDF)

Figure S6 Time courses of cytokine protein expression after administration of TNF-α in vivo. Data points are the means of the median fluorescent intensities resulting from the cytokine assays performed on duodenal lysates, normalized to a loading control dataset on each plate. The color scheme is based on genotype (cyan for wild-type, red for Rag1 null), while the solid and broken lines represent low (5 µg) and high (10 µg) doses of TNF-α, respectively. Error bars represent the SEM for three mice.
(PDF)

Figure S7 Statistical modeling of TNF-α-induced apoptosis in the context of MCP-1 neutralization. (A) PLSDA model-based prediction of the apoptotic phenotype in the duodenum based on phospho-protein signaling data from samples derived from animals treated with TNF-α after pretreatment with anti-MCP-1 (magenta) or non-specific antibody (cyan). The *y*-axis is the numerical result calculated with the PLSDA function of being classified into the “late/low apoptosis” (class 1, cyan) phenotypic class. The red broken line is the threshold that defines classification. Note that the non-specific antibody control classified into class 1. (B) PLSDA model-based prediction of the classification into the “early/low apoptosis” (class 2, blue) phenotypic class with signaling data from duodenal samples from animals treated with TNF-a after pre-treatment with anti-MCP-1 (magenta) or non-specific antibody (cyan). (C) PLSDA model-based prediction of the classification into the “no apoptosis” (class 6, grey) phenotypic class with signaling data from duodenal samples from animals treated with TNF-α after pre-treatment with anti-MCP-1 (magenta) or non-specific antibody (cyan).
(PDF)

Figure S8 Evaluation of exogenous MCP-1 activity and confirmation of immune cell ablation. (A) Evaluation of MCP-1 restoration in Rag1 null animals. A single injection of 2.5 µg of recombinant murine MCP-1 restores of intestinal level to that of wild-type mice. (B) Effect of exogenous MCP-1 on TNF-α-induced apoptosis in Rag1 null animals. Exogenous MCP-1 fails to protect. (C) FACS plots demonstrating ablation of pDCs in Rag1 null animals by treating with anti-PDCA1 (20 mg/kg) for 2 d. (D) Validation of cell depletion experiments. Natural killer cells were depleted by pretreating mice with anti-NK1.1 (12 mg/kg) for 2 d. pDCs were depleted by treating mice with anti-PDCA1 (20 mg/kg) for 2 d. Macrophages were depleted by treating animals with clodronate liposomes (250 µl) for 2 d. In all experiments, error bars represent the SEM for three mice.
(PDF)

Figure S9 Early TNF-α-induced signaling is modulated by plasmacytoid dendritic cells. Early signaling (0.5 h) induced by TNF-α in the duodenum as measured by Bio-Plex phospho-protein signal assays for wild-type mice (cyan), wild-type mice pretreated with anti-MCP-1 for 2 h (magenta), Rag1 null mice (red), and Rag1 null mice pretreated with anti-PDCA-1 for 2 d (yellow). Data are normalized to a loading control dataset on each plate. Error bars represent the SEM for three mice.
(PDF)

Figure S10 Early cytokine induction by TNF-α is modulated by plasmacytoid dendritic cells. Early cytokine protein levels (0.5 h) induced by TNF-α (5 µg) in the duodenum as measured by Bio-Plex cytokine assays for wild-type mice (cyan), wild-type mice pretreated with anti-MCP-1 for 2 h (magenta), Rag1 null mice (red), and Rag1 null mice pretreated with anti-PDCA-1 for 2 d (yellow). Data are normalized to a loading control dataset on each plate. Error bars represent the SEM for three mice.
(PDF)

## Abbreviations

CC3: cleaved caspase 3
CCR2: C-C chemokine receptor 2
DBZ: dibenzazepine
IFN-γ: interferon gamma
Ighm: Immunoglobulin heavy chain mu
ILCs: innate lymphoid cells
LV: latent variable
MAdCAM: mucosal addressin cell adhesion molecule 1
MCP-1: monocyte chemo-attractant protein 1
NK: natural killer
pDCs: plasmacytoid dendritic cells
PH3: phosphorylated histone H3
PLSDA: partial least squares discriminant analysis
Rag: Recombination activating gene
ROC: receiver operating characteristic
TCR: T cell receptor
Th: T helper
TNF-α: tumor necrosis factor alpha
Treg: T regulatory
WT: wild-type
