# Correction: Multi-Scale In Vivo Systems Analysis Reveals the Influence of Immune Cells on TNF-α-Induced Apoptosis in the Intestinal Epithelium

**DOI:** 10.1371/annotation/60833838-f1b9-4f9c-ae0b-485c4acd09c1

**Published:** 2012-10-26

**Authors:** Ken S. Lau, Virna Cortez-Retamozo, Sarah R. Philips, Mikael J. Pittet, Douglas A. Lauffenburger, Kevin M. Haigis

The Author Summary section that follows the Abstract is included in the PDF, but is missing from the online version of the article. The text of the Author Summary is as follows.

Author Summary

Homeostasis in the intestine is maintained through regulated interaction between several cell communities: the intestinal epithelium, intestinal immune cells, and the commensal flora living in the gut. A breakdown in the communication network that exists between these three components can lead to a pathologic state, for example inflammation. We have used computational modeling of intracellular and extracellular signaling pathways to determine how intestinal immune cells control the response of intestinal epithelial cells to the inflammatory cytokine tumor necrosis factor alpha (TNF-α). Cycles of computational modeling and experimental perturbation revealed a network in which epithelial cells communicate with the intestinal immune component via monocyte chemoattractant protein 1 (MCP-1) and the immune cells signal back to the epithelium through interferon gamma (IFN-γ). This extracellular signaling pathway modulates the epithelial apoptotic response to TNF-α in order to regain a state of homeostasis. Our study highlights the power of applying systems biology approaches to mouse models in order to characterize complex biological phenomena.

