# Supplementary material for: Multi-Scale In Vivo Systems Analysis Reveals the Influence of Immune Cells on TNF-α-Induced Apoptosis in the Intestinal Epithelium
Source: PLoS Biol. 2012 Sep 25;10(9):e1001393. doi: 10.1371/journal.pbio.1001393 (PMC3463506; doi:10.1371/journal.pbio.1001393)
Supplement: Figure S1 — Effects of lymphocyte perturbation on acute TNF-α-induced apoptosis. (A) An example of a Western blot for caspase 3 cleavage and β-Tubulin in the duodena of Rag1 null mice (left) and wild-type mice (right) as a time course following TNF-α (5 µg) stimulation. (B) Caspase 3 cleavage induced 2 h post-TNF-α (5 µg) administration within the duodena of wild-type control mice (cyan), Rag1 null mice (red), TCRβ/δ (Tcrb/Tcrd) null mice (dark red), and immunoglobulin heavy chain mu (Ighm) null mice (brown). Data are normalized to wild-type control mice and error bars represent SEM for three mice. (C) Quantification of CD8+ T cells in the duodena of wild-type mice by FACS in wild-type controls (clear) or mice pretreated with anti-MadCAM1 (2 mg/kg) for 2 h (shaded). Treatment with anti-MadCAM1 prevents TNF-α-induced recruitment of T cells to the intestine. Error bars represent the SEM for three mice. (D) Caspase 3 cleavage within the duodena of wild-type control mice (white), Rag1 null mice (green), and wild-type mice pretreated with anti-MadCAM1 (shaded). Data are normalized to wild-type control mice and error bars represent the SEM for three mice. (PDF) [file pbio.1001393.s001.pdf]

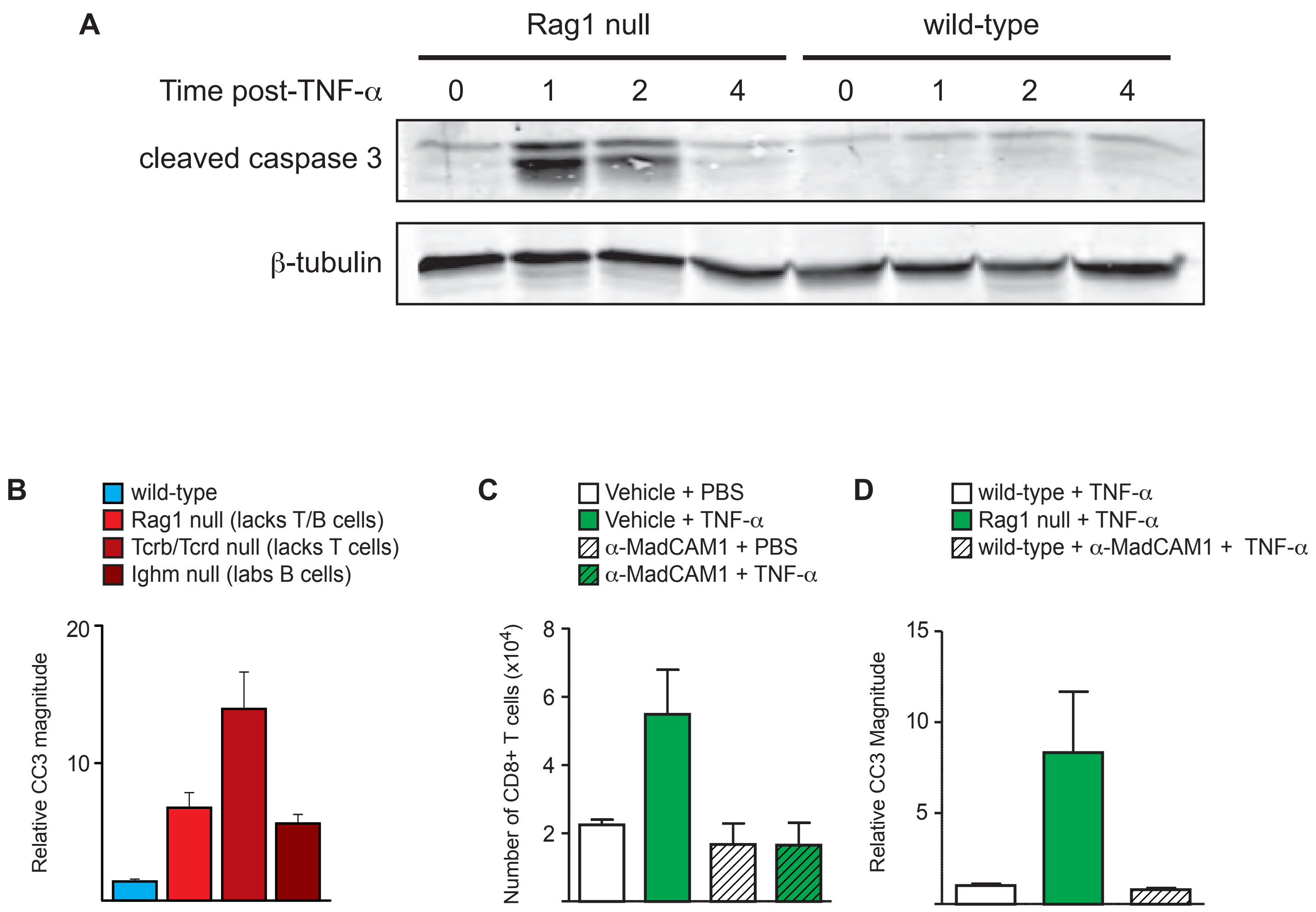

Figure S1. Effects of lymphocyte perturbation on acute TNF-α-induced apoptosis. (A) An example of a western blot for caspase 3 cleavage and β-Tubulin in the duodena of Rag1 null mice (left) and wild-type mice (right) as a time course following TNF-α (5 μg) stimulation. (B) Caspase 3 cleavage induced 2 hours post-TNF-α (5 μg) administration within the duodena of wild-type control mice (cyan), Rag1 null mice (red), TCRβ/δ (Tcrb/Tcrd) null mice (dark red), and immunoglobulin heavy chain mu (Ighm) null mice (brown). Data are normalized to wild-type control mice and error bars represent SEM for 3 mice. (C) Quantification of CD8+ T cells in the duodena of wild-type mice by FACS in wild-type controls (clear) or mice pretreated with anti-MadCAM1 (2 mg/kg) for 2 hours (shaded). Treatment with anti-MadCAM1 prevents TNF-α-induced recruitment of T cells to the intestine. Error bars represent the SEM for 3 mice. (D) Caspase 3 cleavage within the duodena of wild-type control mice (white), Rag1 null mice (green), and wild-type mice pretreated with anti-MadCAM1 (shaded). Data are normalized to wild-type control mice and error bars represent SEM for 3 mice.
