# Supplementary material for: Multi-Scale In Vivo Systems Analysis Reveals the Influence of Immune Cells on TNF-α-Induced Apoptosis in the Intestinal Epithelium
Source: PLoS Biol. 2012 Sep 25;10(9):e1001393. doi: 10.1371/journal.pbio.1001393 (PMC3463506; doi:10.1371/journal.pbio.1001393)
Supplement: Figure S2 — Acute TNF-α-induced apoptosis in the intestine is not affected by modulation of the microbiota. (A) Fecal flora of wild-type mice on normal drinking water or on drinking water supplemented with broad spectrum antibiotics (200 µg/ml ampicillin, 50 µg/ml Primaxin) for 10 d. Fecal flora were plated on MacConkey agar (pink) and Rose agar (red). (B) Time course of caspase 3 cleavage induced by TNF-α as determined by quantitative Western blotting in the duodena of wild-type mice after antibiotic treatment above (broken line) or normal drinking water (solid line). Data are normalized to the peak signal of the wild-type control. Error bars represent the SEM for three mice. (PDF) [file pbio.1001393.s002.pdf]

A

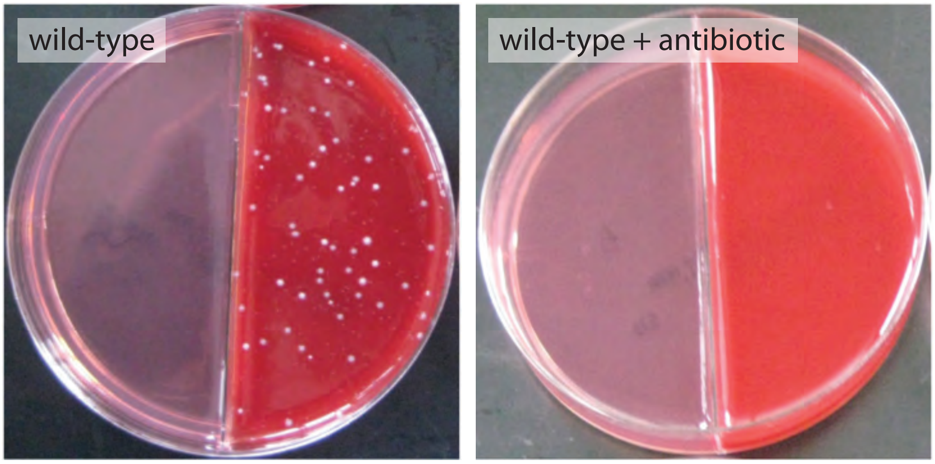

B

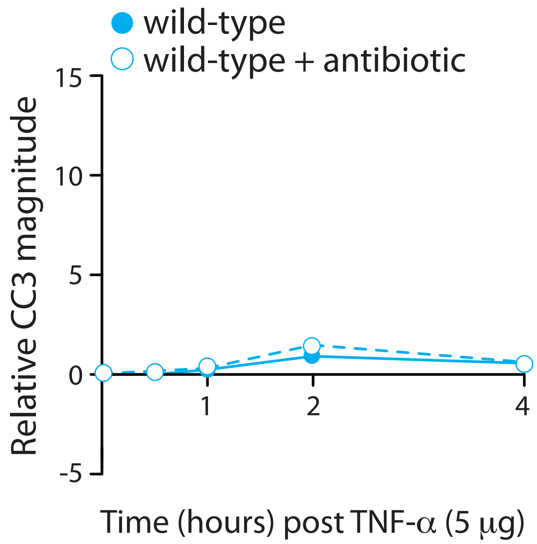

Figure S2. Acute TNF-α-induced apoptosis in the intestine is not affected by modulation of the microbiota. (A) Fecal flora of wild-type mice on normal drinking water or on drinking water supplemented with broad spectrum antibiotics (200 μg/ml ampicillin, 50 μg/ml Primaxin) for 10 days. Fecal flora were plated on MacConkey agar (pink) and Rose agar (red). (B) Time course of caspase 3 cleavage induced by TNF-α as determined by quantitative western blotting in the duodena of wild-type mice after antibiotic treatment above (broken line) or normal drinking water (solid line). Data are normalized to the peak signal of the wild-type control. Error bars represent the SEM for 3 mice.
