# Supplementary material for: Multi-Scale In Vivo Systems Analysis Reveals the Influence of Immune Cells on TNF-α-Induced Apoptosis in the Intestinal Epithelium
Source: PLoS Biol. 2012 Sep 25;10(9):e1001393. doi: 10.1371/journal.pbio.1001393 (PMC3463506; doi:10.1371/journal.pbio.1001393)
Supplement: Figure S3 — Signaling network sampled in our analysis. Here, we present a manually curated network of inflammatory signaling according to canonical pathways. We depict components and connections that are included in our analysis (Input, TNF-α in yellow; Signals, cytokines in light blue, phospho-proteins in dark blue; Response, cleaved caspase 3 in red). Note that additional connections between the nodes likely exist, and note the presence or absence of particular connections that are cell context dependent. (PDF) [file pbio.1001393.s003.pdf]

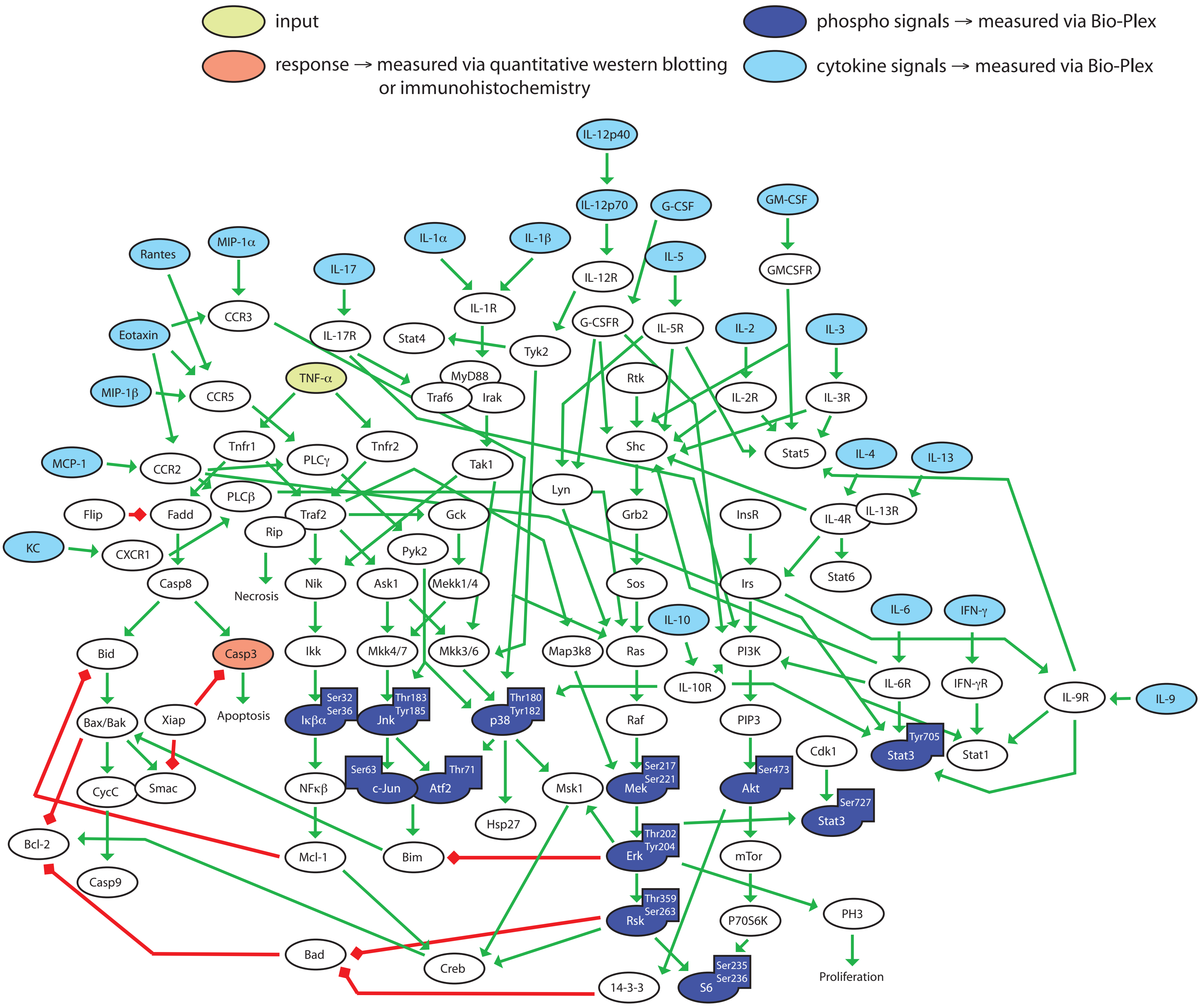

input
response → measured via quantitative western blotting or immunohistochemistry
phospho signals → measured via Bio-Plex
cytokine signals → measured via Bio-Plex
IL-12p40
IL-12p70
G-CSF
GM-CSF
MIP-1α
Rantes
IL-1α
IL-1β
IL-17
IL-12R
IL-5
GMCSFR
CCR3
Eotaxin
IL-1R
G-CSFR
IL-5R
IL-2
IL-3
IL-17R
Stat4
Tyk2
MyD88
TNF-α
MIP-1β
CCR5
Traf6
Irak
Rtk
IL-2R
IL-3R
Tnfr1
Tnfr2
PLCγ
Shc
Stat5
IL-4
IL-13
MCP-1
CCR2
Tak1
Lyn
PLCβ
Flip
Fadd
Traf2
Gck
Grb2
InsR
IL-4R
IL-13R
Rip
KC
CXCR1
Pyk2
Casp8
Necrosis
Nik
Ask1
Mekk1/4
Sos
Irs
Stat6
IL-6
IFN-γ
IL-10
Bid
Casp3
Ikk
Mkk4/7
Mkk3/6
Map3k8
Ras
PI3K
IL-10R
IL-6R
IFN-γR
IL-9R
IL-9
Apoptosis
Bax/Bak
Xiap
Ser32 Ser36
Iκβα
Thr183 Tyr185
Jnk
Thr180 Tyr182
p38
Raf
PIP3
Cdk1
Tyr705
Stat3
Stat1
CycC
Smac
NFκβ
Ser63
c-Jun
Thr71
Atf2
Msk1
Ser217 Ser221
Mek
Ser473
Akt
Ser727
Stat3
Hsp27
Bcl-2
Casp9
Mcl-1
Bim
Thr202 Tyr204
Erk
mTor
Thr359 Ser263
Rsk
P70S6K
PH3
Bad
Creb
14-3-3
Ser235 Ser236
S6
Proliferation

Figure S3. Signaling network sampled in our analysis. Here, we present a manually curated network of inflammatory signaling according to canonical pathways. We depict components and connections that are included in our analysis (Input: TNF-α in yellow, Signals: cytokines in light blue, phospho-proteins in dark blue, Response: cleaved caspase 3 in red). Note that additional connections between the nodes likely exist, and note the presence or absence of particular connections that are cell context dependent.
