# Supplementary material for: Multi-Scale In Vivo Systems Analysis Reveals the Influence of Immune Cells on TNF-α-Induced Apoptosis in the Intestinal Epithelium
Source: PLoS Biol. 2012 Sep 25;10(9):e1001393. doi: 10.1371/journal.pbio.1001393 (PMC3463506; doi:10.1371/journal.pbio.1001393)
Supplement: Figure S4 — Time courses of protein phosphorylation signals activated following exposure to TNF-α in vivo. Data points are the means of the median fluorescent intensities resulting from the phospho-protein assays, normalized to a loading control dataset on each plate. The left side of each graph set contains the duodenal data, while the right side contains the ileal data. The color scheme is based on genotype (cyan for wild-type, red for Rag1 null), while the solid and broken lines represent low (5 µg) and high (10 µg) doses of TNF-α, respectively. Error bars represent the SEM for three mice. (PDF) [file pbio.1001393.s004.pdf]

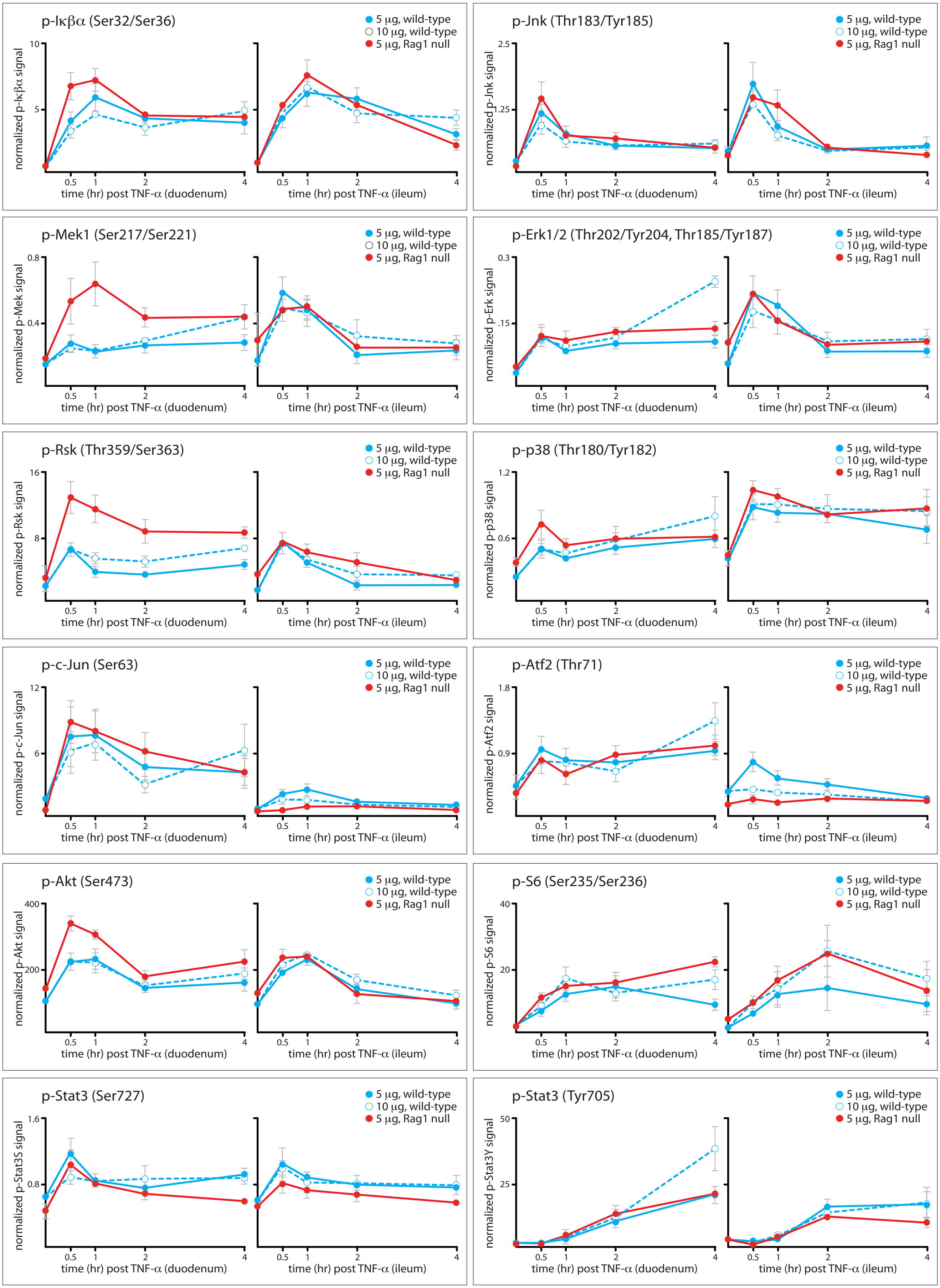
p-Iκβα (Ser32/Ser36)
normalized p-Iκβα signal
p-Jnk (Thr183/Tyr185)
normalized p-Jnk signal
p-Mek1 (Ser217/Ser221)
normalized p-Mek signal
p-Erk1/2 (Thr202/Tyr204, Thr185/Tyr187)
normalized p-Erk signal
p-Rsk (Thr359/Ser363)
normalized p-Rsk signal
p-p38 (Thr180/Tyr182)
normalized p-p38 signal
p-c-Jun (Ser63)
normalized p-c-Jun signal
p-Atf2 (Thr71)
normalized p-Atf2 signal
p-Akt (Ser473)
normalized p-Akt signal
p-S6 (Ser235/Ser236)
normalized p-S6 signal
p-Stat3 (Ser727)
normalized p-Stat3S signal
p-Stat3 (Tyr705)
normalized p-Stat3Y signal
5 µg, wild-type
10 µg, wild-type
5 µg, Rag1 null
time (hr) post TNF-α (duodenum)
time (hr) post TNF-α (ileum)

Figure S4. Time courses of protein phosphorylation signals activated following exposure to TNF-α *in vivo*. Data points are the means of the median fluorescent intensities resulting from the phospho-protein assays, normalized to a loading control dataset on each plate. The left side of each graph set contains the duodenal data while the right side contains the ileal data. The color scheme is based on genotype (cyan for wild-type, red for Rag1 null), while the solid and broken lines represent low (5 μg) and high (10 μg) doses of TNF-α, respectively. Error bars represent SEM for 3 mice.
