# Supplementary material for: Multi-Scale In Vivo Systems Analysis Reveals the Influence of Immune Cells on TNF-α-Induced Apoptosis in the Intestinal Epithelium
Source: PLoS Biol. 2012 Sep 25;10(9):e1001393. doi: 10.1371/journal.pbio.1001393 (PMC3463506; doi:10.1371/journal.pbio.1001393)
Supplement: Figure S5 — Modeling signaling network shifts in modulating TNF-α-induced apoptosis. (A) A 3-D PLSDA model describes dosage, regional, and genotypic effects on TNF-α-induced apoptosis. Error of classification into class 1 (late/low apoptosis, cyan), class 2 (early/low apoptosis, blue), and class 3 (early/high apoptosis, red) of calibration data, using models with increasing numbers of latent variables. The average error across all classes (black) includes the first three classes and the three ileum classes (no apoptosis). Classification error is defined as the probability of misclassifying a sample given the sample distribution and a model. (B) Error of classification into classes 1, 2, and 3 of data kept out of the cross-validation procedure, using models built with the rest of the data, with increasing numbers of latent variables. The average error across all classes (black) includes the first three classes and the three ileum classes (no apoptosis). Note that the average error is greater than the error for individual classes due to the fact that the ileum classes (no apoptosis) are very similar at the signaling and phenotypic level and cannot be distinguished from one another. (C) Receiver operating characteristic (ROC) curves depicting specificity (1-false positive rate) versus sensitivity (true positive rate) as the numerical threshold for classification is changed. The quality of a classification model can be evaluated by the degree by which the curves deviate from the center diagonal, with more deviation describing a better classification. ROC curves for the six classes using a PLSDA with three LVs. The red circle represents the discriminant threshold selected for a model. (D) Loadings on LV3, the latent variable for describing the timing of apoptosis. The y-axis quantifies the positive or negative contribution of a particular signal to LV3. Late signals correlate to an early apoptotic phenotype, consistent with our previous findings. (PDF) [file pbio.1001393.s005.pdf]

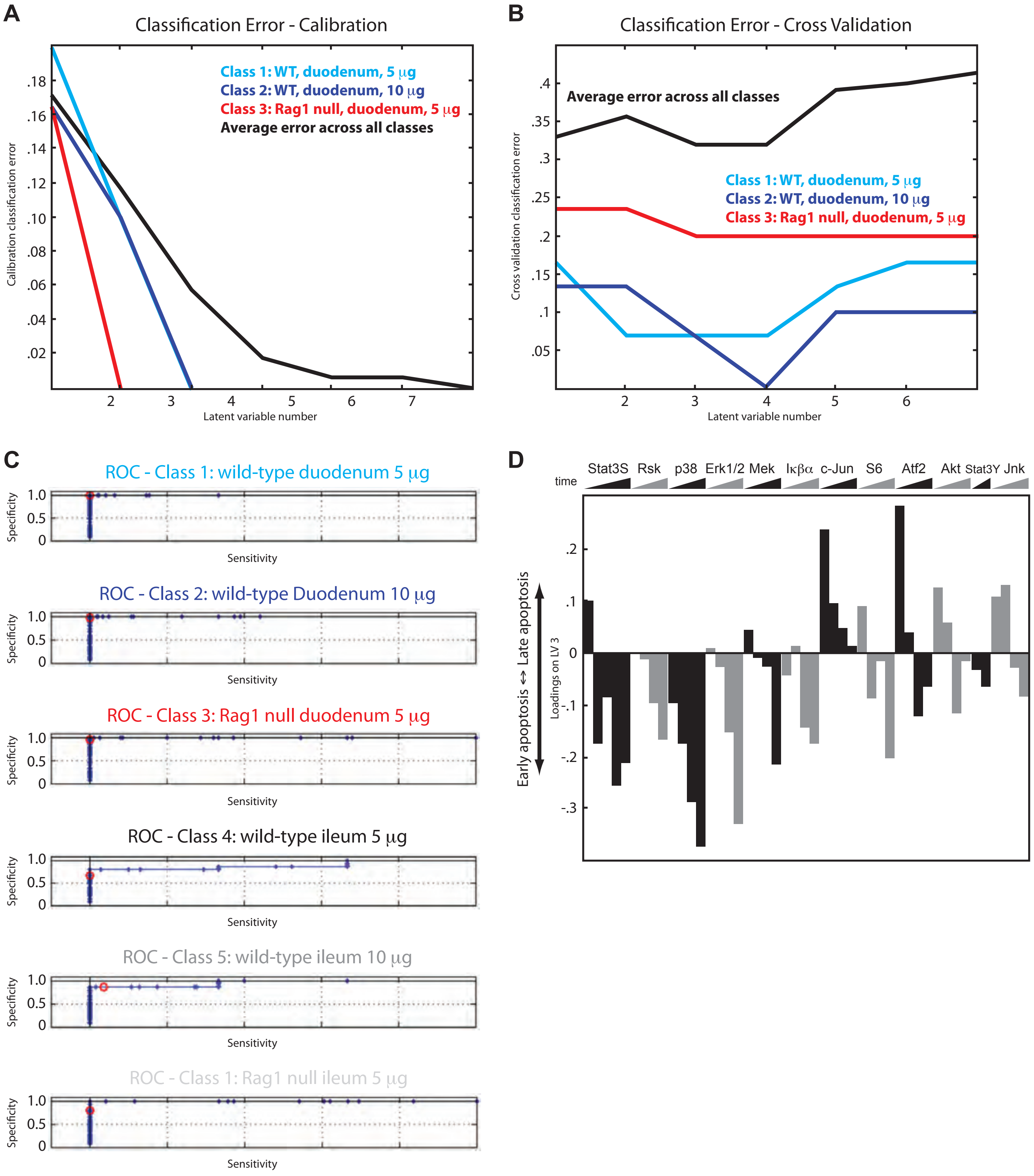
A
Classification Error - Calibration
Class 1: WT, duodenum, 5 µg
Class 2: WT, duodenum, 10 µg
Class 3: Rag1 null, duodenum, 5 µg
Average error across all classes
Calibration classification error
Latent variable number
B
Classification Error - Cross Validation
Average error across all classes
Class 1: WT, duodenum, 5 µg
Class 2: WT, duodenum, 10 µg
Class 3: Rag1 null, duodenum, 5 µg
Cross validation classification error
Latent variable number
C
ROC - Class 1: wild-type duodenum 5 µg
ROC - Class 2: wild-type Duodenum 10 µg
ROC - Class 3: Rag1 null duodenum 5 µg
ROC - Class 4: wild-type ileum 5 µg
ROC - Class 5: wild-type ileum 10 µg
ROC - Class 1: Rag1 null ileum 5 µg
Specificity
Sensitivity
D
time
Stat3S Rsk p38 Erk1/2 Mek Iκβα c-Jun S6 Atf2 Akt Stat3Y Jnk
Early apoptosis ↔ Late apoptosis
Loadings on LV 3

Figure S5. Modeling signaling network shifts in modulating TNF-α-induced apoptosis. (A) A 3D PLSDA model describes dosage, regional, genotypic effects on TNF-α-induced apoptosis. Error of classification into class 1 (late/low apoptosis, cyan), class 2 (early/low apoptosis, blue), and class 3 (early/high apoptosis, red) of calibration data, using models with increasing numbers of latent variables. The average error across all classes (black) includes the first three classes and the three ileum classes (no apoptosis). Classification error is defined as the probability of misclassifying a sample given the sample distribution and a model. (B) Error of classification into classes 1, 2, and 3 of data kept out of the cross-validation procedure, using models built with the rest of the data, with increasing numbers of latent variables. The average error across all classes (black) includes the first three classes and the three ileum classes (no apoptosis). Note that the average error is greater than the error for individual classes due to the fact that the ileum classes (no apoptosis) are very similar at the signaling and phenotypic level and cannot be distinguished from one another. (C) Receiver operating characteristic (ROC) curves depicting specificity (1-false positive rate) versus sensitivity (true positive rate) as the numerical threshold for classification is changed. The quality of a classification model can be evaluated by the degree by which the curves deviate from the center diagonal, with more deviation describing a better classification. ROC curves for the 6 classes using a PLSDA with 3 LVs. The red circle represents the discriminant threshold selected for a model. (D) Loadings on LV3, the latent variable for describing the timing of apoptosis. The Y axis quantifies the positive or negative contribution of a particular signal to LV3. Late signals correlate to an early apoptotic phenotype, consistent with our previous findings.
