# Supplementary material for: Multi-Scale In Vivo Systems Analysis Reveals the Influence of Immune Cells on TNF-α-Induced Apoptosis in the Intestinal Epithelium
Source: PLoS Biol. 2012 Sep 25;10(9):e1001393. doi: 10.1371/journal.pbio.1001393 (PMC3463506; doi:10.1371/journal.pbio.1001393)
Supplement: Figure S6 — Time courses of cytokine protein expression after administration of TNF-α in vivo. Data points are the means of the median fluorescent intensities resulting from the cytokine assays performed on duodenal lysates, normalized to a loading control dataset on each plate. The color scheme is based on genotype (cyan for wild-type, red for Rag1 null), while the solid and broken lines represent low (5 µg) and high (10 µg) doses of TNF-α, respectively. Error bars represent the SEM for three mice. (PDF) [file pbio.1001393.s006.pdf]

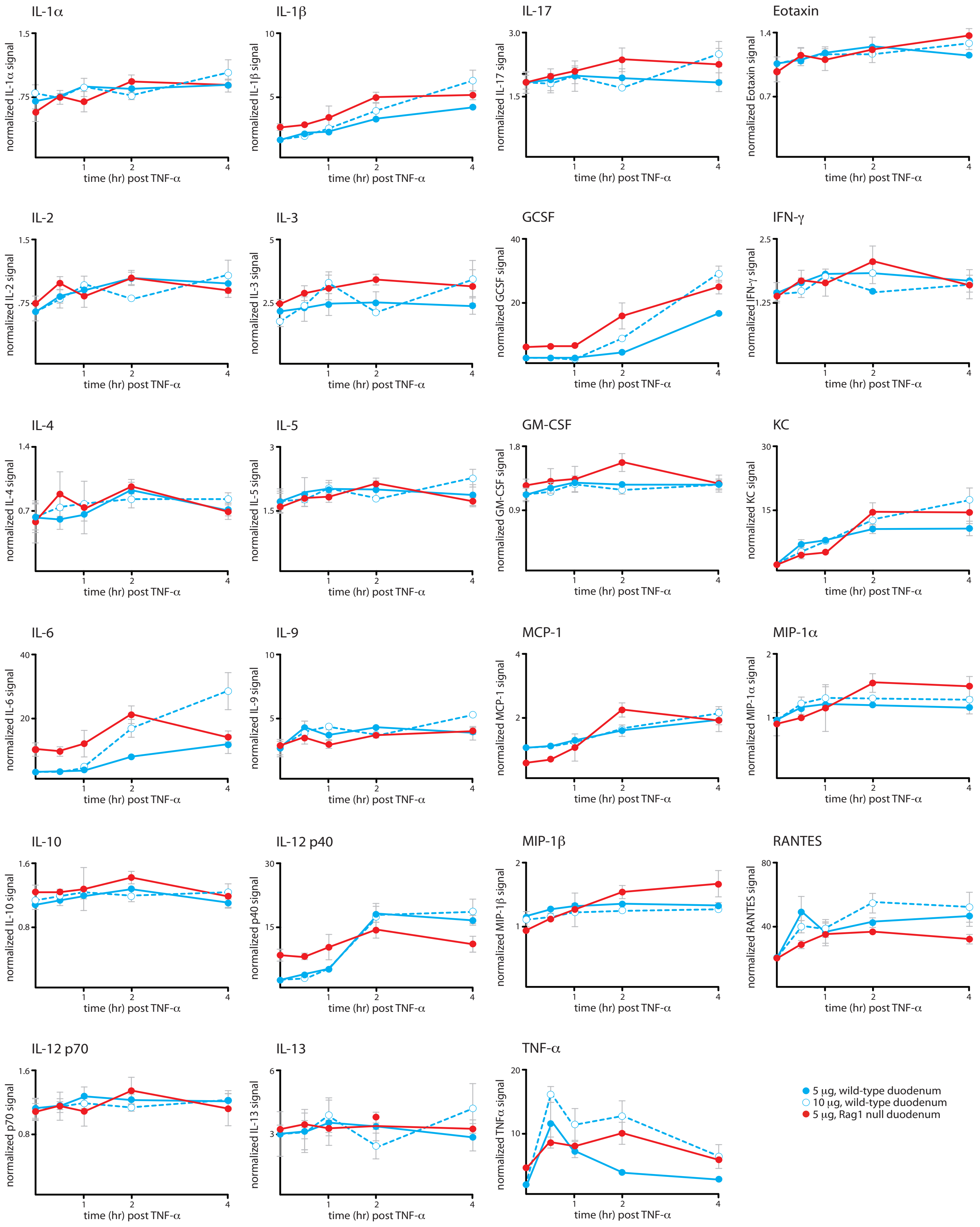

IL-1α
normalized IL-1α signal
1.5
.75
time (hr) post TNF-α
IL-1β
normalized IL-1β signal
10
5
time (hr) post TNF-α
IL-17
normalized IL-17 signal
3.0
1.5
time (hr) post TNF-α
Eotaxin
normalized Eotaxin signal
1.4
0.7
time (hr) post TNF-α
IL-2
normalized IL-2 signal
1.5
.75
time (hr) post TNF-α
IL-3
normalized IL-3 signal
5
2.5
time (hr) post TNF-α
GCSF
normalized GCSF signal
40
20
time (hr) post TNF-α
IFN-γ
normalized IFN-γ signal
2.5
1.25
time (hr) post TNF-α
IL-4
normalized IL-4 signal
1.4
0.7
time (hr) post TNF-α
IL-5
normalized IL-5 signal
3
1.5
time (hr) post TNF-α
GM-CSF
normalized GM-CSF signal
1.8
0.9
time (hr) post TNF-α
KC
normalized KC signal
30
15
time (hr) post TNF-α
IL-6
normalized IL-6 signal
40
20
time (hr) post TNF-α
IL-9
normalized IL-9 signal
10
5
time (hr) post TNF-α
MCP-1
normalized MCP-1 signal
4
2
time (hr) post TNF-α
MIP-1α
normalized MIP-1α signal
2
1
time (hr) post TNF-α
IL-10
normalized IL-10 signal
1.6
0.8
time (hr) post TNF-α
IL-12 p40
normalized p40 signal
30
15
time (hr) post TNF-α
MIP-1β
normalized MIP-1β signal
2
1
time (hr) post TNF-α
RANTES
normalized RANTES signal
80
40
time (hr) post TNF-α
IL-12 p70
normalized p70 signal
1.6
0.8
time (hr) post TNF-α
IL-13
normalized IL-13 signal
6
3
time (hr) post TNF-α
TNF-α
normalized TNFα signal
20
10
time (hr) post TNF-α
1
2
4
5 μg, wild-type duodenum
10 μg, wild-type duodenum
5 μg, Rag1 null duodenum

Figure S6. Time courses of cytokine protein expression after administration of TNF-α *in vivo*. Data points are the means of the median fluorescent intensities resulting from the cytokine assays performed on duodenal lysates, normalized to a loading control dataset on each plate. The color scheme is based on genotype (cyan for wild-type, red for Rag1 null), while the solid and broken lines represent low (5 μg) and high (10 μg) doses of TNF-α, respectively. Error bars represent SEM for 3 mice.
