# Supplementary material for: Multi-Scale In Vivo Systems Analysis Reveals the Influence of Immune Cells on TNF-α-Induced Apoptosis in the Intestinal Epithelium
Source: PLoS Biol. 2012 Sep 25;10(9):e1001393. doi: 10.1371/journal.pbio.1001393 (PMC3463506; doi:10.1371/journal.pbio.1001393)
Supplement: Figure S7 — Statistical modeling of TNF-α-induced apoptosis in the context of MCP-1 neutralization. (A) PLSDA model-based prediction of the apoptotic phenotype in the duodenum based on phospho-protein signaling data from samples derived from animals treated with TNF-α after pretreatment with anti-MCP-1 (magenta) or non-specific antibody (cyan). The y-axis is the numerical result calculated with the PLSDA function of being classified into the “late/low apoptosis” (class 1, cyan) phenotypic class. The red broken line is the threshold that defines classification. Note that the non-specific antibody control classified into class 1. (B) PLSDA model-based prediction of the classification into the “early/low apoptosis” (class 2, blue) phenotypic class with signaling data from duodenal samples from animals treated with TNF-a after pre-treatment with anti-MCP-1 (magenta) or non-specific antibody (cyan). (C) PLSDA model-based prediction of the classification into the “no apoptosis” (class 6, grey) phenotypic class with signaling data from duodenal samples from animals treated with TNF-α after pre-treatment with anti-MCP-1 (magenta) or non-specific antibody (cyan). (PDF) [file pbio.1001393.s007.pdf]

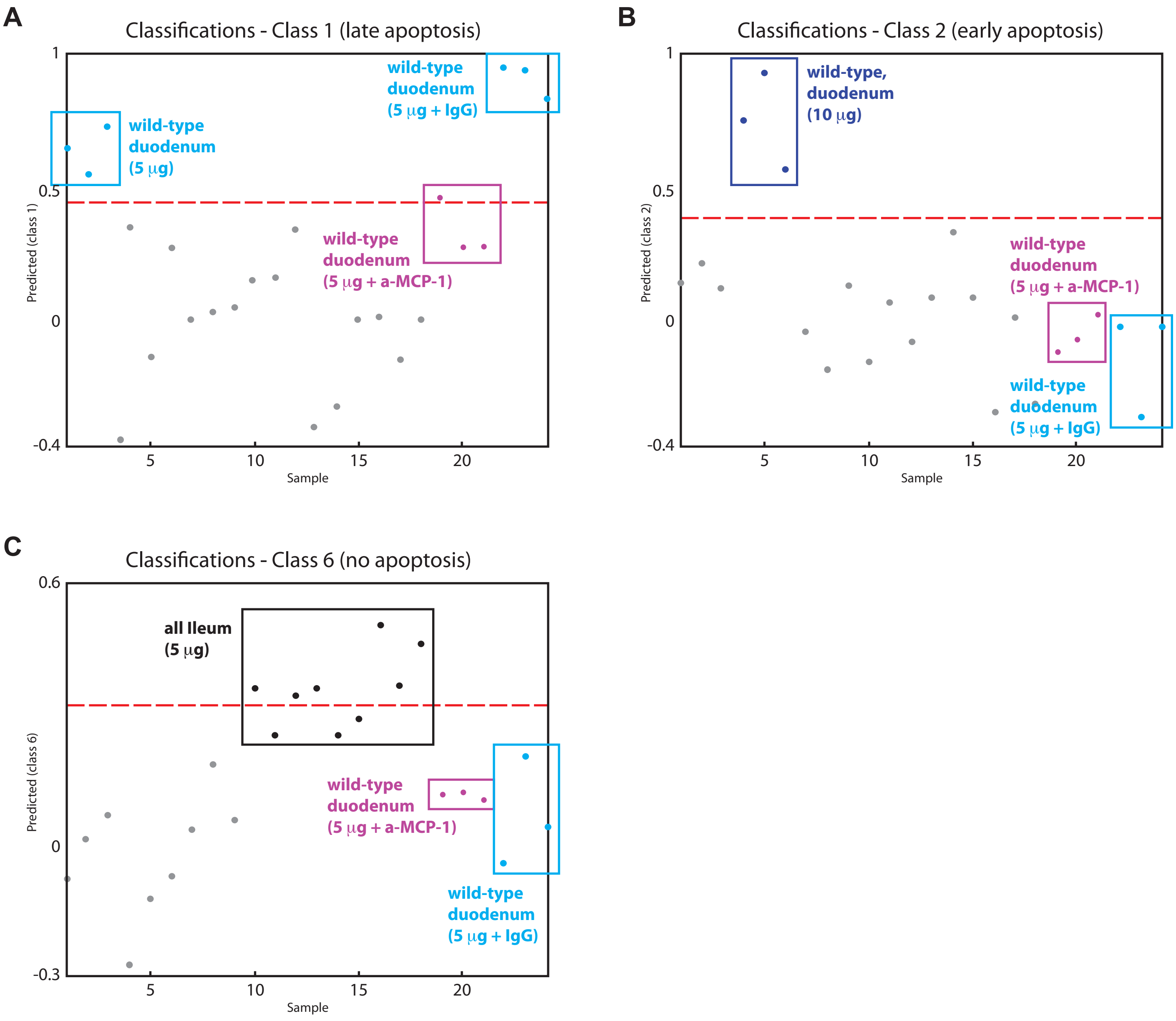


**Figure S7. Statistical modeling of TNF-α-induced apoptosis in the context of MCP-1 neutralization.** (A) PLSDA model–based prediction of the apoptotic phenotype in the duodenum based on phospho-protein signaling data from samples derived from animals treated with TNF-α after pretreatment with anti-MCP-1 (magenta) or non-specific antibody (cyan). The y-axis is the numerical result calculated with the PLSDA function of being classified into the “late/low apoptosis” (class 1 - cyan) phenotypic class. The red broken line is the threshold that defines classification. Note that the non-specific antibody control classified into class 1. (B) PLSDA model–based prediction of the classification into the “early/low apoptosis” (class 2 - blue) phenotypic class with signaling data from duodenal samples from animals treated with TNF-α after pre-treatment with anti-MCP-1 (magenta) or non-specific antibody (cyan). (C) PLSDA model–based prediction of the classification into the “no apoptosis” (class 6 - grey) phenotypic class with signaling data from duodenal samples from animals treated with TNF-α after pre-treatment with anti-MCP-1 (magenta) or non-specific antibody (cyan).
