# Supplementary material for: Multi-Scale In Vivo Systems Analysis Reveals the Influence of Immune Cells on TNF-α-Induced Apoptosis in the Intestinal Epithelium
Source: PLoS Biol. 2012 Sep 25;10(9):e1001393. doi: 10.1371/journal.pbio.1001393 (PMC3463506; doi:10.1371/journal.pbio.1001393)
Supplement: Figure S8 — Evaluation of exogenous MCP-1 activity and confirmation of immune cell ablation. (A) Evaluation of MCP-1 restoration in Rag1 null animals. A single injection of 2.5 µg of recombinant murine MCP-1 restores of intestinal level to that of wild-type mice. (B) Effect of exogenous MCP-1 on TNF-α-induced apoptosis in Rag1 null animals. Exogenous MCP-1 fails to protect. (C) FACS plots demonstrating ablation of pDCs in Rag1 null animals by treating with anti-PDCA1 (20 mg/kg) for 2 d. (D) Validation of cell depletion experiments. Natural killer cells were depleted by pretreating mice with anti-NK1.1 (12 mg/kg) for 2 d. pDCs were depleted by treating mice with anti-PDCA1 (20 mg/kg) for 2 d. Macrophages were depleted by treating animals with clodronate liposomes (250 µl) for 2 d. In all experiments, error bars represent the SEM for three mice. (PDF) [file pbio.1001393.s008.pdf]

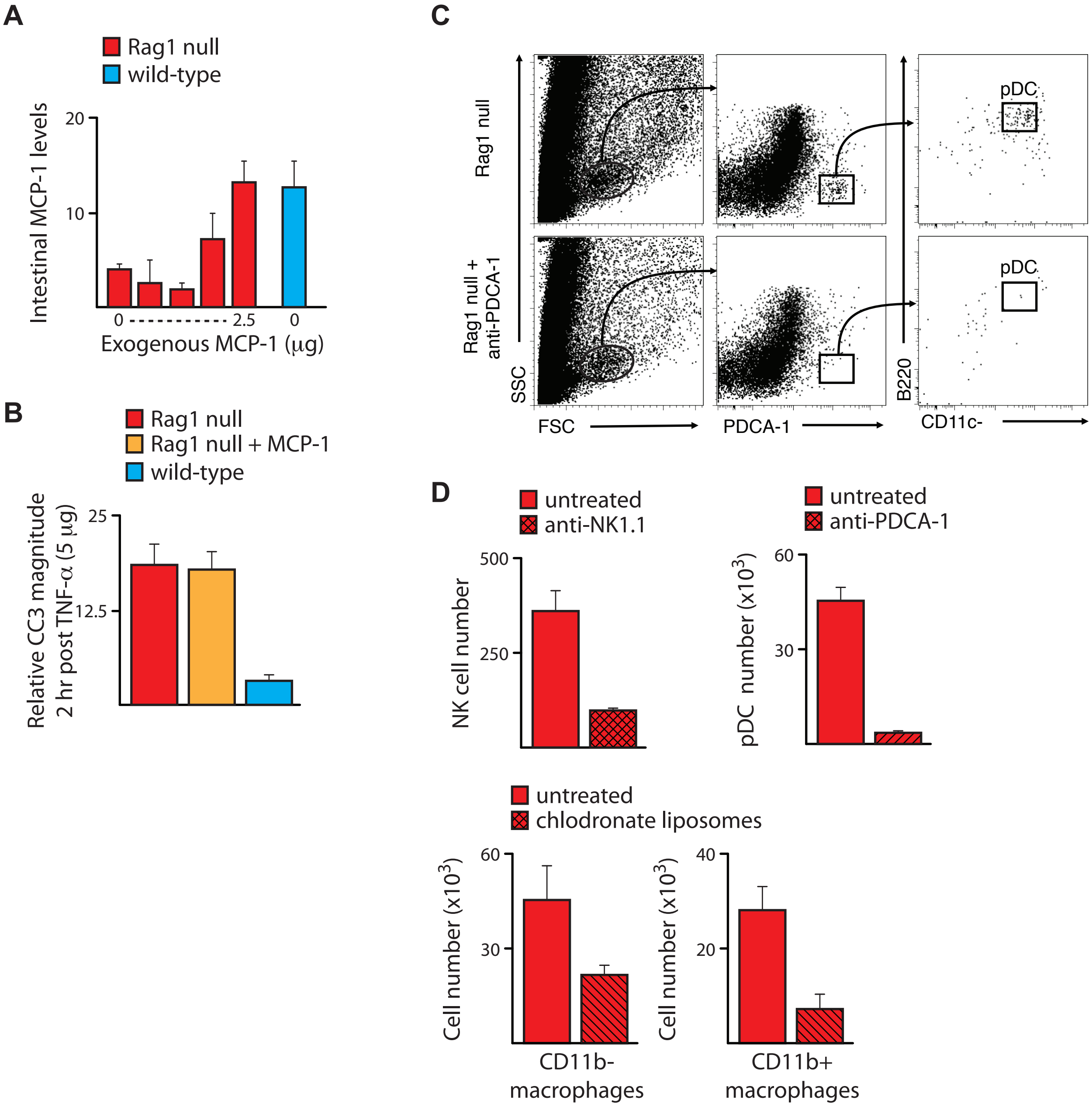

Figure S8. Evaluation of exogenous MCP-1 activity and confirmation of immune cell ablation. (A) Evaluation of MCP-1 restoration in Rag1 null animals. A single injection of 2.5 μg of recombinant murine MCP-1 restores of intestinal level to that of wild-type mice. (B) Effect of exogenous MCP-1 on TNF-α-induced apoptosis in Rag1 null animals. Exogenous MCP-1 fails to protect. (C) FACS plots demonstrating ablation of pDCs in Rag1 null animals by treating with anti-PDCA1 (20 mg/kg) for 2 days. (D) Validation of cell depletion experiments. Natural killer cells were depleted by pretreating mice with anti-NK1.1 (12 mg/kg) for 2 days. pDCs were depleted by treating mice with anti-PDCA1 (20 mg/kg) for 2 days. Macrophages were depleted by by treating animals with clodronate liposomes (250 μl) for 2 days. In all experiments, error bars represent the SEM for 3 mice.
