# Supplementary material for: Multi-Scale In Vivo Systems Analysis Reveals the Influence of Immune Cells on TNF-α-Induced Apoptosis in the Intestinal Epithelium
Source: PLoS Biol. 2012 Sep 25;10(9):e1001393. doi: 10.1371/journal.pbio.1001393 (PMC3463506; doi:10.1371/journal.pbio.1001393)
Supplement: Figure S9 — Early TNF-α-induced signaling is modulated by plasmacytoid dendritic cells. Early signaling (0.5 h) induced by TNF-α in the duodenum as measured by Bio-Plex phospho-protein signal assays for wild-type mice (cyan), wild-type mice pretreated with anti-MCP-1 for 2 h (magenta), Rag1 null mice (red), and Rag1 null mice pretreated with anti-PDCA-1 for 2 d (yellow). Data are normalized to a loading control dataset on each plate. Error bars represent the SEM for three mice. (PDF) [file pbio.1001393.s009.pdf]

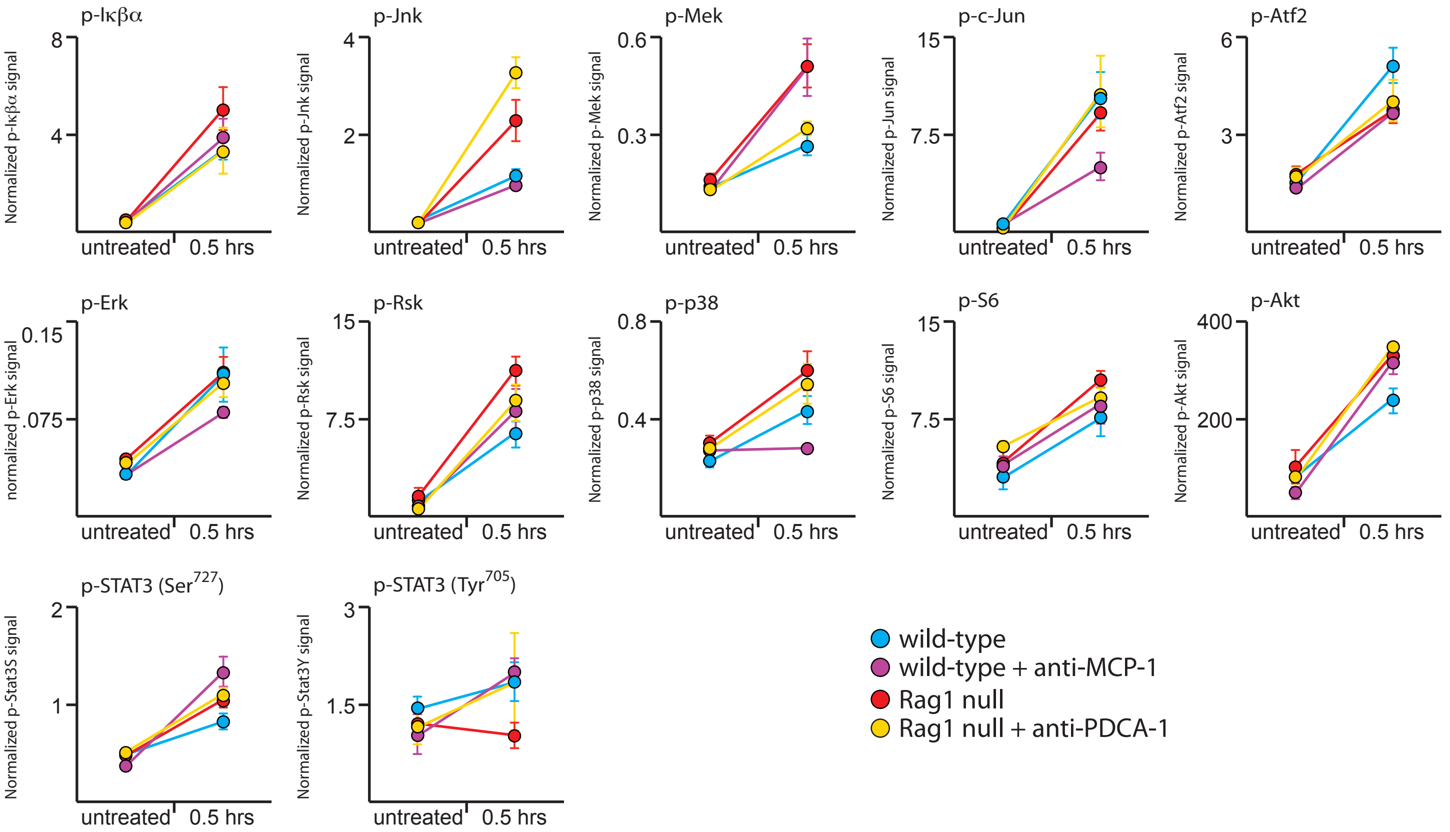

Figure S9. Early TNF-α-induced signaling is modulated by plasmacytoid dendritic cells. Early signaling (0.5 h) induced by TNF-α in the duodenum as measured by Bio-Plex phospho-protein signal assays for wild-type mice (cyan), wild-type mice pretreated with anti-MCP-1 for 2 hours (magenta), Rag1 null mice (red), and Rag1 null mice pretreated with anti-PDCA-1 for 2 days (yellow). Data are normalized to a loading control dataset on each plate. Error bars represent SEM for 3 mice.
